# Sequence Assembly of *Yarrowia lipolytica* Strain W29/CLIB89 Shows Transposable Element Diversity

**DOI:** 10.1371/journal.pone.0162363

**Published:** 2016-09-07

**Authors:** Christophe Magnan, James Yu, Ivan Chang, Ethan Jahn, Yuzo Kanomata, Jenny Wu, Michael Zeller, Melanie Oakes, Pierre Baldi, Suzanne Sandmeyer

**Affiliations:** 1 Department of Computer Science, School of Computer Sciences, University of California Irvine, Irvine, California, United States of America; 2 Institute for Genomics and Bioinformatics, University of California Irvine, Irvine, California, United States of America; 3 Department of Biological Chemistry, School of Medicine, University of California Irvine, Irvine, California, United States of America; University of Strasbourg, FRANCE

## Abstract

*Yarrowia lipolytica*, an oleaginous yeast, is capable of accumulating significant cellular mass in lipid making it an important source of biosustainable hydrocarbon-based chemicals. In spite of a similar number of protein-coding genes to that in other Hemiascomycetes, the *Y*. *lipolytica* genome is almost double that of model yeasts. Despite its economic importance and several distinct strains in common use, an independent genome assembly exists for only one strain. We report here a *de novo* annotated assembly of the chromosomal genome of an industrially-relevant strain, W29/CLIB89, determined by hybrid next-generation sequencing. For the first time, each *Y*. *lipolytica* chromosome is represented by a single contig. The telomeric rDNA repeats were localized by Irys long-range genome mapping and one complete copy of the rDNA sequence is reported. Two large structural variants and retroelement differences with reference strain CLIB122 including a full-length, novel Ty3/Gypsy long terminal repeat (LTR) retrotransposon and multiple LTR-like sequences are described. Strikingly, several of these are adjacent to RNA polymerase III-transcribed genes, which are almost double in number in *Y*. *lipolytica* compared to other Hemiascomycetes. In addition to previously-reported dimeric RNA polymerase III-transcribed genes, tRNA pseudogenes were identified. Multiple full-length and truncated LINE elements are also present. Therefore, although identified transposons do not constitute a significant fraction of the *Y*. *lipolytica* genome, they could have played an active role in its evolution. Differences between the sequence of this strain and of the existing reference strain underscore the utility of an additional independent genome assembly for this economically important organism.

## Introduction

The oleaginous yeast *Yarrowia lipolytica* is an industrial model organism for production of biosustainable hydrocarbon-based chemicals [1–6]. *Y*. *lipolytica* is one of the most divergent of the characterized Hemiascomycetes [7]. Despite a genome almost twice the size of *Saccharomyces cerevisiae*, *Y*. *lipolytica* is not thought to have undergone whole genome duplication [8]. In addition, *Y*. *lipolytica* has more traits in common with metazoan cells than other characterized yeasts. These include dispersed 5S genes, signal-recognition-particle type 7SL RNA sequence, and a greater fraction of the genome composed of introns and intergenic sequences [7, 8]. The *Y*. *lipolytica* genome also contains representatives of diverse classes of transposable elements, including remnants of a DNA transposon [9], long-terminal repeat (LTR) [10] and non-LTR *L*ong *IN*terspersed *E*lement (LINE) [11] retrotransposons [12]. Finally, unlike the more widely-studied respiro-fermentative *S*. *cerevisiae*, *Y*. *lipolytica* is an obligate aerobe. It metabolizes a wide range of carbon substrates including lipids, paraffins, oils, glycerol, and acetate and is capable of accumulating a high percentage of cell weight in lipid [1, 13, 14]. This metabolism has recently been tuned for production of hydrocarbon chemicals.

Availability of an annotated, complete genome assembly is a significant advantage for the study of any organism. The current *Y*. *lipolytica* genomic reference sequence, YALI0, is that of *Y*. *lipolytica* strain E150/CLIB122 (hereafter CLIB122) [7, 8, 15] (http://www.ncbi.nlm.nih.gov/genome/genomes/194). The YALI0 assembly features the six chromosomes that have been reduced to thirteen contigs and genes that have been extensively annotated [reviewed [8]]. CLIB122 was derived from a cross between isolates from a Paris sewer (W29/CLIB89, hereafter CLIB89) and an American corn processing plant (CBS6124-2) [16]. Some current strains of industrial interest, including PO1f [17], were derived directly from CLIB89 [8, 13, 18]. Draft reference genomes of *Y*. *lipolytica* PO1f of 348 contigs [19] and CLIB89 of 369 contigs [20] have recently been assembled by alignment with the CLIB122 assembly. However, a complete and independent assembly of strain CLIB89 has been lacking.

We report here the *de novo* assembly and annotation of the *Y*. *lipolytica* strain CLIB89 genome. Illumina and PacBio sequencing enabled a hybrid assembly of single contigs for chromosomes A-F and mitochondrial chromosome M. Irys long-range genome mapping was utilized to identify extensions of rDNA repeats on the left ends of chromosomes A, C, and F and the right end of chromosome B. Complete sequences of key genetic markers, *URA3* and *LEU2*, and one copy of rDNA sequence not represented in the CLIB122 annotation, were determined. A potentially active copy of Tyl3, a Ty3/Gypsy-like long terminal repeat (LTR) retrotransposon was discovered. Comparison of the sequence of the CLIB89 genome to related strain CLIB122 revealed unexpected differences in numbers and types of transposable elements.

## Results and Discussion

### Assembly and annotation

#### Genome assembly

**Fig 1:**
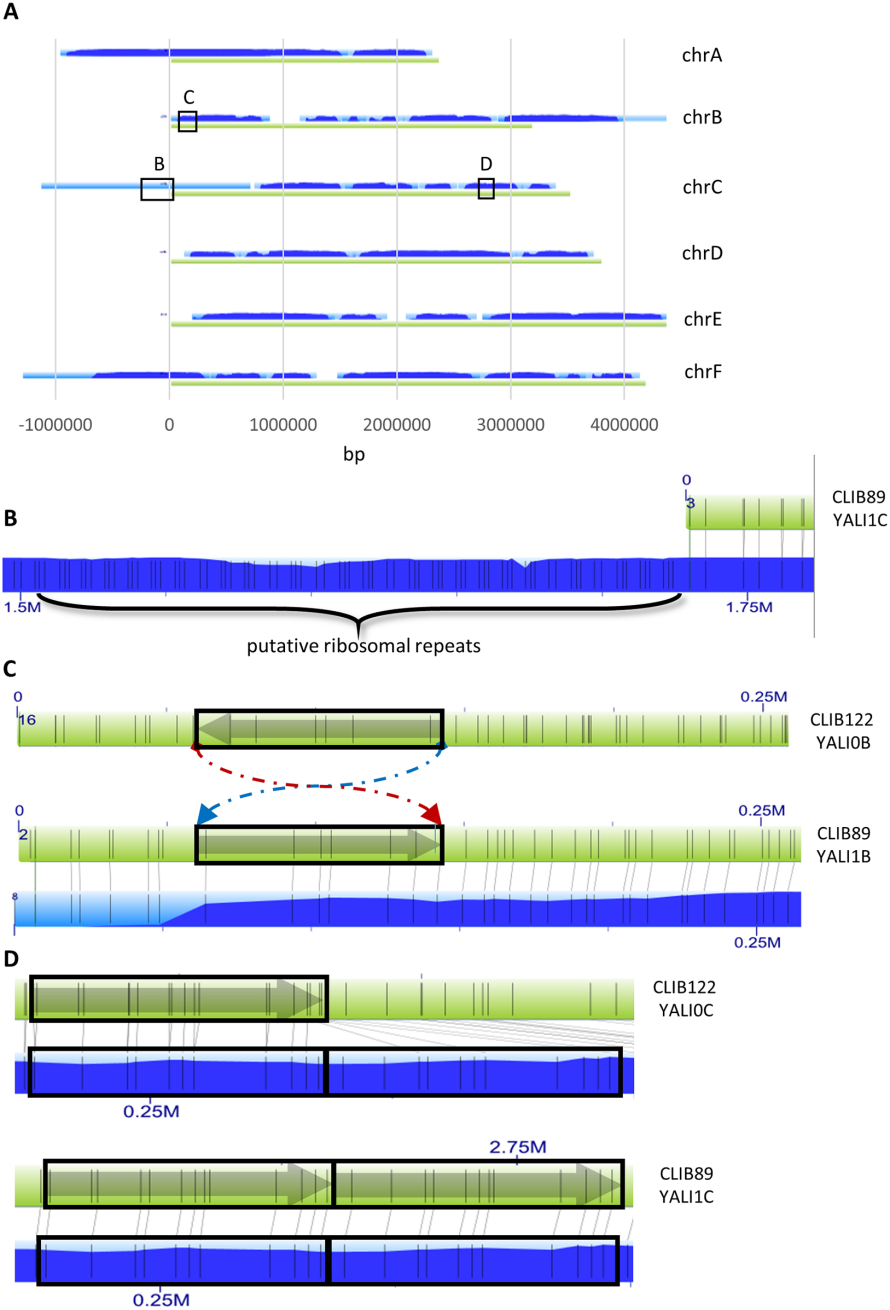
BioNano Irys long-range mapping of CLIB89 YALI1 and comparison to CLIB122 YALI0. (**A**) Irys molecules assembled into contigs (coverage indicated as light and dark for lesser and greater coverage, respectively) aligned with the six CLIB89 YALI1 chromosomes (green) show extensions in four chromosome terminal regions. (**B**) Chromosomal extensions show a repeated pattern of Nt.BspQI nickase sites (vertical grey lines), consistent with tandem, copies of ribosomal (r) RNA-coding sequence. (**C**) and (**D**) Alignment of map of Nt.BspQI sites in the CLIB122 YALI0 (upper green bar) and CLIB89 YALI1 assembly (lower green bar) with the physical Nt.BspQI map generated by Irys technology (blue bar) shows they differ by a 71-kb inversion on chromosome B (**C**) and a 54-kb repeat on chromosome C in CLIB89 YALI1 (**D**).

The *Y*. *lipolytica* genome sequence was determined by HiSeq 2500 (Illumina Inc.) and PacBio RS II (Pacific Biosciences) high-throughput sequencing coupled to a hybrid assembly pipeline (Materials and Methods, Table 1 and S1 Text). First, overlapping short, high-quality Illumina HiSeq 2500 sequencing reads were merged into contigs; second, long PacBio reads were used to traverse retrotransposons and bridge the HiSeq contigs, and third, junctions were further refined by aligning with high-quality Illumina reads. PCR was used to confirm key contig junctions (S1 Table). In the next phase, the Irys long-range genome mapping system (BioNano Genomics Inc.) was used to evaluate the integrity of the Illumina-PacBio hybrid assembly, estimate the extent of unassembled sequence in telomeric regions, and localize rDNA repeats (Materials and Methods, Fig 1, Table 2). The CLIB89 genome assembly was designated YALI1 to distinguish it from the previous CLIB122 YALI0 assembly (previously http://www.genolevures.org/index.html#; CLIB122 YALI0 is now maintained at http://gryc.inra.fr and at http://www.ncbi.nlm.nih.gov/genome/genomes/194) [7]. Initial comparison of CLIB89 YALI1 and CLIB122 YALI0 assemblies showed that they were similar in both individual chromosomal lengths as well as in total chromosome lengths (Table 3). The total length is also similar to that of the draft sequences of the CLIB89-derived PO1f strain of 19,922,824 bp [19] and to that of the CLIB89 20.3 Mb draft sequence [20]. Both of those were assembled by alignment to the CLIB122 YALI0 reference sequence.

**Table 1:** CLIB89 YALI1 sequence read statistics.

Dataset	Platform	Reads	Read length	Average coverage
YL97B	SR HiSeq 2000	14,951,623	97	69
YL110	PR HiSeq 2500	389,608,406	110	2041
YLP13	PacBio RS II	157,966	3362	25
YLP14	PacBio RS II	253,645	5642	68

**Table 2:** Irys CLIB89 YALI1 assembly.

Number of consensus genome scaffolds	31
Consensus genome scaffold size (Mb)	25.2
Number of molecules mapped	621,169
Mapped molecule quantity (Mb)	40,798.6
Mapped average size (kb)	269
Average depth of molecule coverage	71.3
Average label density (per 100 kb)	14.4

**Table 3:** Chromosome assembly lengths.

Chromosome	CLIB122 (nts)	CLIB89 (nts)
A	2,303,261	2,257,857
B	3,066,374	3,044,971
C	3,272,609	3,366,276
D	3,633,272	3,629,463
E	4,224,103	4,198,534
F	4,003,362	4,002,965
**Total**	**20,502,981**	**20,500,066**
M	47,916	47,926

#### Genome annotation

Fungal genomes differ from some other metazoan genomes in having a high density of coding sequences, low frequency of introns, and in some in codon usage. These differences combined with the medical and agricultural importance of fungi have motivated development of fungal specific analysis strategies. In order to preserve any significant differences between the genomes, the CLIB89 assembly was performed independently of the previous CLIB122 assembly, and identified significant differences from CLIB122. The existence of a previously annotated genome for *Y*. *lipolytica* CLIB122 was an important asset in this project. This reference genome was used for comparison to identified genes and gene-naming. However, the CLIB89 assembly is the first in which each chromosome is represented by a single contig.

**Fig 2:**
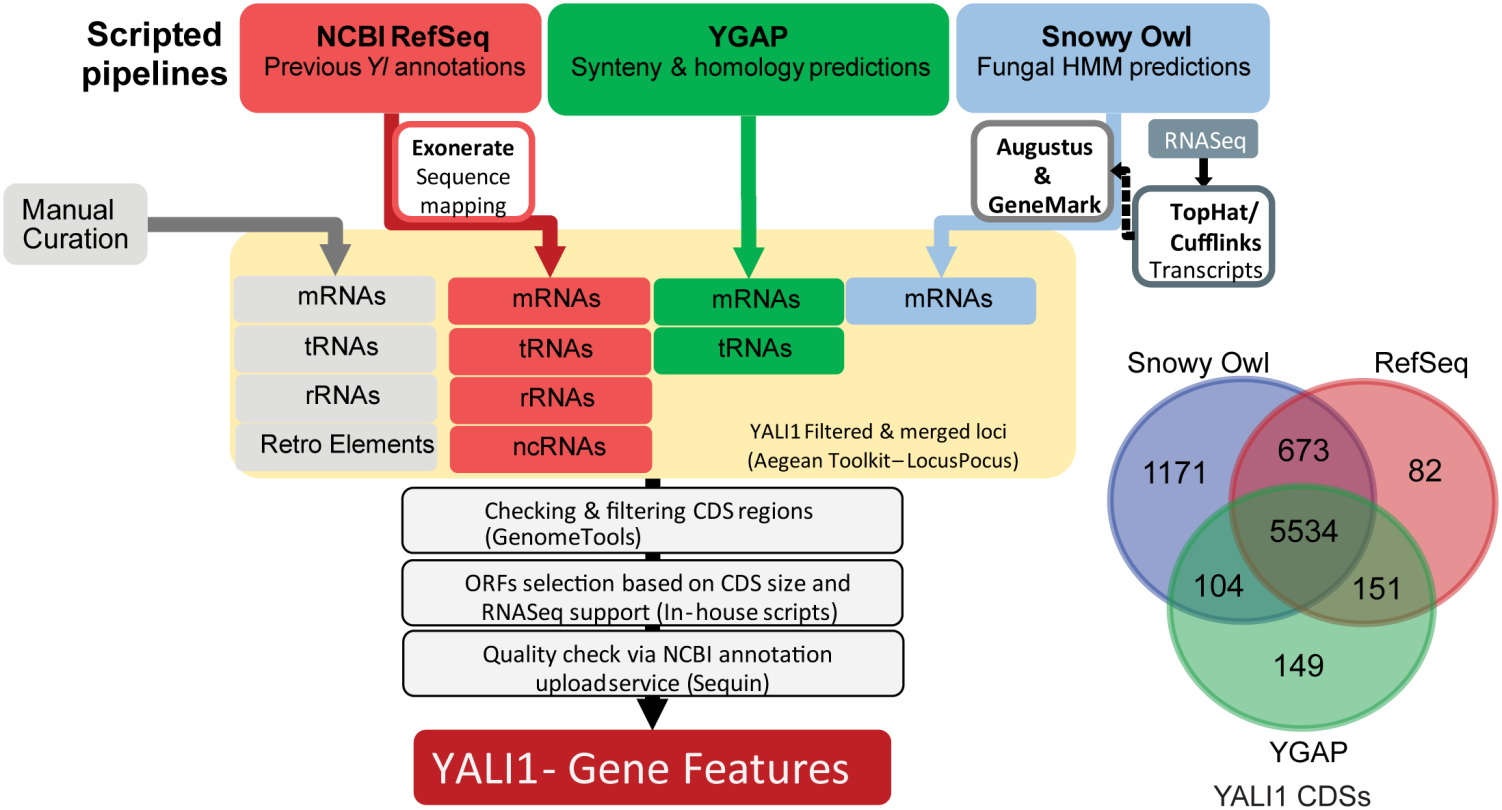
*Y*. *lipolytica* CLIB89 YALI1 *a*nnotation pipeline. YALI1 Annotations were derived from a combination of three automated annotation pipelines and a set of manual Blast searches. The three pipelines consist of mapping existing *Yl* annotations from CLIB122 YALI0 (NCBI RefSeq) to the CLIB89 YALI1 sequence; synteny and homology predictions (YGAP); and fungal HMM predictions (Snowy Owl). Loci of identified features were merged, checked for consistency, selected for CDS based on size and RNA-Seq support, and vetted through NCBI's Sequin upload service to produce the final set of gene features. Contributions and agreements for CDS features from the three automated pipelines are shown in the Venn diagram to the lower right.

**Fig 3:**
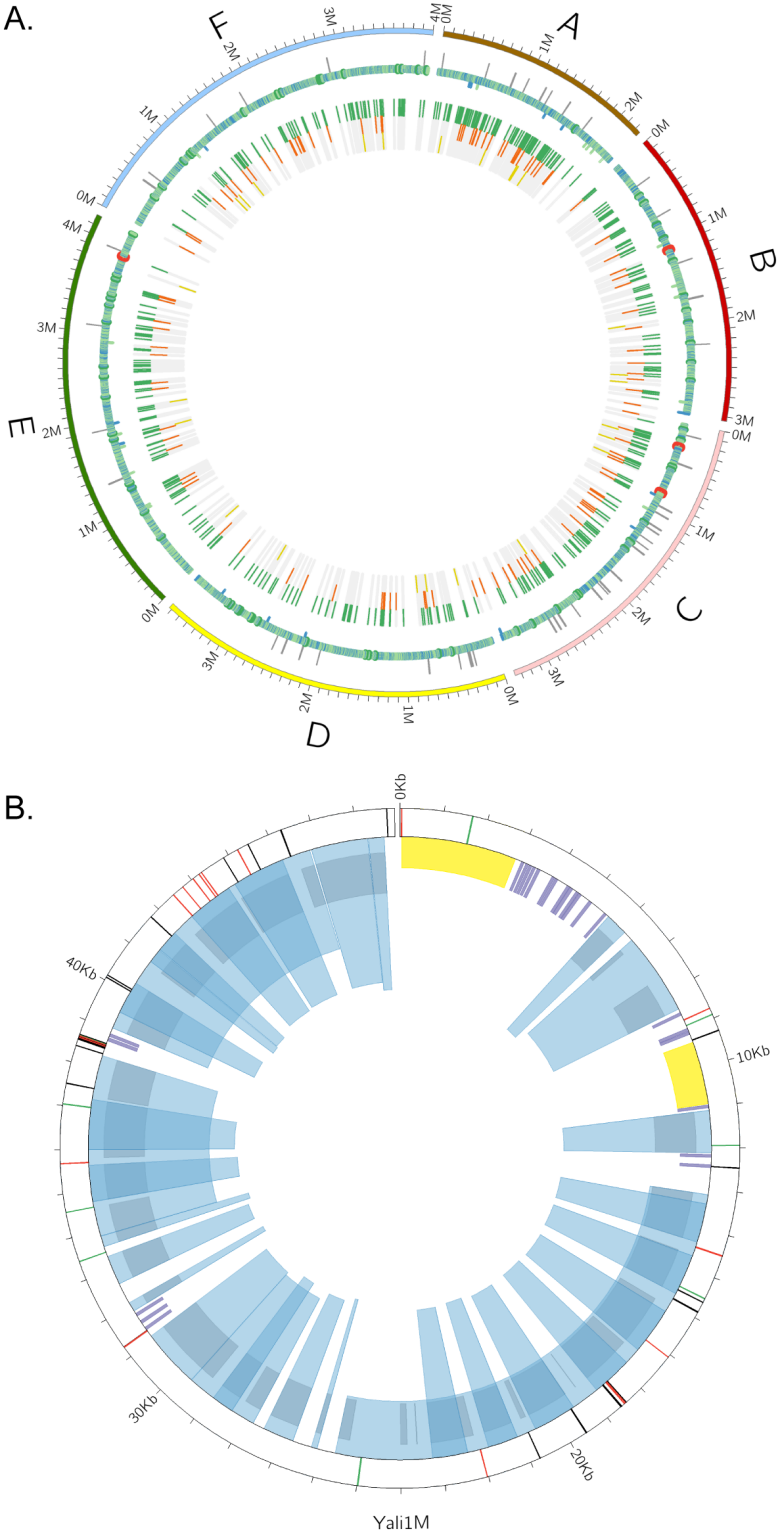
CIRCOS overview of YALI1 gene features. **(A)** Chromosomal genes. Outer ring, chromosomes. First mapping track [RNA polymerase II (POL2)-transcribed genes]: LINE retroelements (outward light grey posts), overlapping genes on both strands (inward blue posts), POL2 less than 1 kb (blue rings), POL2 between 1 kb and 5 kb (light green rings), POL2 between 5 kb and 10 kb (green rings), and POL2 > 10 kb (red rings). The next inner track (POL3 and POL2 ncRNA genes), tRNA (green), rRNA (orange), and ncRNA genes (dark yellow). **(B)** Mitochondrial genes. Transcripts from exons (longer spanning light blue wedges); transcripts from introns (narrower and taller overlapping wedges); CDS (gray); tDNA (purple); and rDNA (yellow). Outer track shows variants comparison with the CLIB122 assembly (http://www.ncbi.nlm.nih.gov/nuccore/NC_002659.1): mismatches (black posts); insertions (green) and deletions (red) relative to the CLIB122 assembly.

The CLIB89 genome was analyzed and annotated using a combination of parallel pipelines: 1) a customized in-house to search for sequences present in the NCBI *Y*. *lipolytica* database (http://www.ncbi.nlm.nih.gov/); 2) Yeast Genome Annotation Pipeline (YGAP) [21]; and 3) SnowyOwl fungal genome analysis [22] (Materials and Methods, Fig 2). The results of this analysis are summarized in Fig 3 and Table 4. Subsequent to this primary analysis, multiple comparisons were made between CLIB89 YALI1 and CLIB122 YALI0 genomes.

**Table 4:** CLIB89/CLIB122 gene content.

	CLIB122^2^	CLIB89^2^	Unique CLIB122	Identified In CLIB89^5^
**mRNA**^1^^,^^2^	6472 CDS	7864 CDS +118 pseudo	31	1428 CDS+88 pseudo
**tDNA YALI [A-F]**^3^	510+9 pseudo	509+9 pseudo	5	5
**tDNA YALI [M]**	26	24	3	1
**5S rDNA [A-F]**	117	111+3+4* pseudo	9	9
**Other ncDNA**^4^	12+13 Ruf70	15+13 Ruf70		3
**Total features**	7150+9 pseudo	8536+134 pseudo	48	1534

^1^RefSeq; reported as CLIB122 “proteins”; CLIB122 (http://www.ncbi.nlm.nih.gov/genome/genomes/194); identified in CLIB89 are CDS identified in the pipelines as described in Materials and Methods; parallel analysis was not performed on CLIB122

^2^Pseudo refers to pseudogenes that resemble original genes, but have interrupted coding sequences

^3^ Eight tDNA-like sequences present in both strains but not previously reported for CLIB122 are included and not counted as unique to CLIB89

^4^ Thirteen Ruf70 present in both assemblies but not annotated in CLIB122

^5^ Annotated in CLIB89 YALI1, but not in CLIB122 YALI0

* Pseudo rRNA too short to include in NCBI annotation

Overall there is high consistency between the CLIB89 YALI1 genome reported here and the previous CLIB122 YALI0 assembly. The NCBI annotated data from *Y*. *lipolytica* is largely based on the CLIB122 assembly [7] (http://www.ncbi.nlm.nih.gov/genome/genomes/194), but also includes other *Y*. *lipolytica* sequences. We refer here to these annotated data as the RefSeq Yl (database) although it is not formally a single curated RefSeq genome sequence. From the protein coding sequences in CLIB122 RefSeq Yl, 31 had no sequence matches to CLIB89 YALI1, while 6,582 matches were found in CLIB89 YALI1 using Exonerate software [23]. After filtering and combining with other annotation pipelines, the RefSeq mapping annotated 6,440 CDS out of the combined total of 7864 CDS, with 82 CDS unique to this pipeline (Fig 2).

For additional gene discovery, CLIB89 YALI1 was analyzed using YGAP software [21]. YGAP was developed for fungal genomes and exploits the existence of a large number of gene sequences (“pillars”) conserved among fungal species and maintained in the Yeast Gene Order Browser (YGOB) database [24] as well as the syntenic arrangement of coding regions among a large number of fungi.

Availability of RNA-seq data from a parallel study in our laboratory enabled SnowyOwl, a fungal Hidden-Markov model (HMM) gene predictor approach that uses transcriptome data for model validation [22]. The SnowyOwl pipeline leveraged a combination of RNA-Seq and gene homology searches for *ab initio* gene prediction.

#### Final merged set of annotated genes

The three sets of gene loci and a handful of manually curated loci were merged into one final set based on common coordinates in the CLIB89 YALI1 assembly. For each protein-coding gene, an optimal ORF configuration was defined based on the best agreement among the three pipelines. The locus ID numbering in YALI0 was revised in YALI1 to accommodate additional sequences assembled at the ends of chromosomes. Common names based on *S*. *cerevisiae* genes and utilized in the YALI0 annotation were retained. Sequences not previously identified as genes were designated following standard nomenclature for Hemiascomycete yeasts and consistent with CLIB122 nomenclature. [Four capital letters refer to the genus and species, one digit to the assembly, A to F and M to the chromosomes and five digits to the coding regions followed by g for proteins, t for repeated sequences, r for non-coding sequences, and s for cis-acting features [7, 25].] The CLIB89 YALI1 coding sequences mapping to CLIB122 YALI0 and annotations, together with YALI1 designations for mRNA, noncoding (nc) and retrotransposon sequences and corresponding YALI0 designations are shown in the S2 Table. Thirty-one genes were identified as unique to CLIB89 (S5 Table). The annotated assembly is available through a browser interface (http://sbsngsserver.biochem.uci.edu/jbrowse/index.html), and has been uploaded to the NCBI website (http://www.ncbi.nlm.nih.gov/). Details of this process are provided in Materials and Methods. Annotations of CLIB89 features are available for uploading and viewing in a genome browser (S3 and S4 Tables) (http://gmod.org/wiki/GFF3).

### Genomic features

#### Chromosomal terminal sequences and rDNA

Four chromosomes were previously reported to contain terminal clusters of rDNA sequence [8, 26, 27]. Nonetheless, the complete rDNA 35S sequence was not determined in either the original CLIB89 YALI1 or CLIB122 YALI0 assemblies. BLAST analysis utilizing *S*. *cerevisiae RDN18-1* sequence to query CLIB122 YALI0 sequence identified a small segment of the rDNA sequence near the end of chromosome F with rDNA similarity. Primers (S1 Table) complementary to sequence near the end of CLIB89 chromosome F and to sequence containing the CLIB122 rDNA segment were used to amplify the intervening DNA from CLIB89 DNA. This sequence contained rDNA sequence thus positioning one copy relative to CLIB89 YALI1 sequence. An overlapping sequence was recovered from high fold coverage unmapped reads from the CLIB89 YALI1 sequence and used to design primers that enabled amplification of an almost full-length copy of the rDNA repeat and adjacent sequences. The products of these PCR reactions were analyzed by Sanger sequencing. BLAST analysis of the assembled sequence showed sequence consistent with the order External Transcribed Spacer (ETS), 18S rRNA, Internal Transcribed Spacer 1 (ITS), 5.8S, ITS 2, 25S rRNA, and flanking NTS sequences (S2 Text). Previous analysis of the *Y*. *lipolytica* rDNA locus showed that the NTS may be present in forms of different lengths [27]. On average, the fold coverage of Illumina sequences was approximately 100X greater than that for uniquely-aligned reads.

Informed by the complete rDNA sequence, a virtual digest with Irys nickase Nt.BspQI was performed to determine the rDNA pattern predicted to be generated by this enzyme. The Nt.BspQI predicted rDNA pattern was compared to the Nt.BspQI pattern of telomeric regions where Irys *Y*. *lipolytica* molecules extended beyond the assembly. This comparison identified predicted rDNA pattern repeats localized to the left ends of chromosomes A, C, and F and the right end of chromosome B (Fig 1B). This result corroborates previous proposals that the rDNA of *Y*. *lipolytica* is comprised of distributed telomere-proximal clusters, and for the first time provides a molecular map of the number and location of these clusters.

In most eukaryotes, maintenance of chromosomal ends is through telomerase 3’ terminal extension using a short guide RNA. The updated *Y*. *lipolytica* telomeric sequence (5’GGGTTAGTCA3’) [8] matched highly-repeated, unassembled reads in our CLIB89 sequences. Due to the repeated nature of these sequences and the absence of Nt.BspQI nickase sites, they could not be assembled into the complete CLIB89 YALI1 genomic sequence.

#### Small non-coding RNA genes

RNA Polymerase III-transcribed genes (RNAP3 genes) in *Y*. *lipolytica* have interesting features that distinguish them from RNAP3 genes in other yeasts [7, 28–30]. First, there are about twice as many; second, the 5S genes are dispersed throughout the genome, rather than clustered within rDNA repeats; and third, dimeric RNAP3 genes are abundant. RNAP3 genes are described in Table 4 and S6 Table. Dimeric genes are characterized by RNAP3 gene-coding sequences separated by only 5 to 26 nts with the upstream member followed by an abbreviated RNAP3 terminator tract of T’s.

From RefSeq Yl mapping, 510 CLIB122 YALI0 tDNA sequences from chromosomes A-F were identified in CLIB89 YALI1. In addition, nine other tDNA-like sequences that did not pass tRNAscan-SE, but had high similarity to tDNA from RefSeq YI and were not annotated in CLIB122 YALI0, were identified as allelic in the two genomes (Table 4, S6 Table). These additional nine genes were classified as tDNA pseudogenes. RNAP3 genes have multiple interactions with retroelements and we speculate that these pseudogenes arose as reverse transcript cDNAs of tRNAs that were integrated into the genome.

A significant fraction of RNAP3 genes are present in multiple copies. In *S*. *cerevisiae* the 5S rDNA occurs within spacers between tandem rDNA repeats. However, in *Y*. *lipolytica* and most metazoan species, the 5S genes are dispersed outside the rDNA repeats [27, 29, 31]. One hundred and eleven 5S genes and seven pseudo 5S genes were identified in CLIB89 of which 103 were perfectly matched to CLIB89 5S genes (Table 4, S6 Table, YALI1 5S gene summary). Of the seven pseudo 5S genes, four were below the length threshold to be submitted to NCBI.

Analysis of the CLIB89 YALI1 genome using Exonerate/BLAST identified single representatives of the RNAP3 *SNR52*, *SCR1*, *SNR6*, and *RPR1* genes and thirteen copies of *RUF70*, as previously annotated in CLIB122 YALI0 [28–30]. Similar to CLIB122 YALI0, copies of Ruf70 were downstream of tDNA^Trp^(CCA) in CLIB89 YALI1. In addition, genes encoding RNAP2-transcribed processing RNAs U1-U5 and U7 RNAs were identified.

### Differences between CLIB89 and CLIB122 genomes

#### Structural variation

CLIB89 YALI1 and CLIB122 YALI0 genomes were globally compared to themselves and to each other by alignment of the assemblies using a dot matrix program, MUMmer (http://mummer.sourceforge.net/) (S1 Fig). Self-alignment highlighted, as expected, repeated sequences throughout the two genomes. Many of these were 5–6 kb or 300–500 bp in length, consistent with the sizes of full-length or truncated LINE elements and LTR retrotransposons or solo LTRs. However, consistent with the Irys map (Fig 1C) comparison of the CLIB89 and CLIB122 assemblies revealed a 71-kb sequence in CLIB89 YALI1 chromosome B that was inverted relative to the same sequence in the CLIB122 YALI0 assembly and the draft CLIB89-related assemblies, PO1f and W29 [7, 19, 32]. PCR was performed across the upstream and downstream junctions of the inversion using primer pairs JY5118/JY5119 and JY5120/JY5121 respectively (S2 Fig). These reactions generated products consistent with the predicted sizes from the CLIB89 YALI1 assembly of 752 bp and 763 bp, respectively. Products with an identical migration pattern were generated when PO1f genomic DNA was used as a template. Furthermore, although a negative result, primer pairs JY5118/JY5121 and JY5119/5420, failed to generate a product, contrary to what was predicted in the CLIB122 YALI0 assembly. This result is consistent with the close relationship of CLIB89 and PO1f.

MUMmer analysis also revealed a striking ~115-kb region in CLIB89 YALI1, but not CLIB122 YALI0. Examination of the DNA sequence in this region showed that, consistent with the Irys map (Fig 1D), a 54-kb sequence was repeated with three 6.5-kb Ylli LINE copies: flanking upstream and downstream and separating the 54-kb repeats. In order to validate this structural variant, PCR was performed using primers JY5124/JY5125 (S1 Table, S2 Fig), complementary to sequences at the downstream and upstream junctions of the 54-kb sequence with the central Ylli sequence. PCR generated a product consistent with the size predicted for an amplicon containing Ylli sequence bounded by head and tail ends of flanking copies of the 54-kb sequence. In the CLIB122 YALI0 assembly, the 54-kb block occurs as a single copy flanked by Ylli sequence direct repeats. These differences between the two assemblies could have been generated by unequal crossing over between the first and second Ylli elements. Local amplification of gene copy number such as this might have advantages for the host. In this case, it is difficult to speculate. There were a total of 29 features including 27 CDS among them genes implicated in stress resistance.

IrysView software was used to align the physical CLIB89 Irys Nt.BspQI molecules and virtual sequence based CLIB89 YALI1 Nt.BspQI patterns. This comparison showed consistency between the physical molecular and sequence maps, thereby confirming the orientation of a 71 kb sequence and the existence of the 54-kb direct repeat flanked by Ylli element fragments in the CLIB89 YALI1 sequence (panels C and D Fig 1).

The CLIB89 YALI1 sequence was examined for the region of chromosome A encompassing four protein-coding genes found in CLIB122, but reported to be absent in the latest PO1f draft assembly (CLIB122: 196442–215157)[19]. This sequence in CLIB122 YALI0 included YALI0A01562 and YALI0A01602, genes encoding proteins with weak similarity to SMC5/6 proteins involved in DNA repair and recombination, leading to speculation that this deficiency contributes to the relatively low ratio of homologous recombination (HR) to non-homologous end joining (NHEJ) observed in *Y*. *lipolytica* [19]. Similarly, our assembly showed that this segment is absent from chromosome A in CLIB89. However, BLAST search of the YALI1 sequence revealed a 93% match to the sequences of YALI0A01562 on chromosome E (YALI1E20467g) and a 95% match to YALI0A01602g on chromosome F (YALI1F03604).

#### Protein-coding differences

After annotation, CLIB89 YALI1 and CLIB122 YALI0 genomes were compared, revealing unique features in each (S2, S5 and S6 Tables). Differences between CLIB89 YALI1 and YALI0 transposons are discussed below. CLIB89 is a wild-type strain so that *URA3* and *LEU2* sequences are represented. However, *URA3* and *LEU2* [13, 18, 33] genes were deleted and disrupted, respectively, in CLIB122 to enable the intact genes to be used as selectable markers. *URA3* encodes orotidine-5’-phosphate decarboxylase, a central enzyme in the uracil biosynthetic pathway. *LEU2*, encodes beta isopropylmalate dehydrogenase, which is critical for leucine biosynthesis and also an important genetic marker [1, 8, 13, 31]. In CLIB122 the *LEU2* ORF, YALI0C00407g, is disrupted by insertion of the *S*. *cerevisiae SUC2* gene for invertase, which allows for growth on sucrose [34]. *Y*. *lipolytica* is thought to be heterothallic. Therefore, rather than switching expression of a mating-type locus, strains themselves are of alternative Mating Types, A and B. CLIB89 possesses the Mating-Type A protein and CLIB122 possesses the Mating-Type B protein, consistent with the previously reported mating types [35]. Twelve additional genes were identified in terminal regions of the chromosomes in the CLIB89 assembly that were not present in the CLIB122 assembly (S5 Table). The simplest explanation is that rather than constituting an actual difference between the strains, this region was incomplete in the CLIB122 YALI0 assembly. These regions contained: one heat-shock gene on chromosome A; a block of seven genes at the end of chromosome D, and four genes within the first 32 kb of chromosome E. This interpretation is supported by sequence reads related to argininosuccinate synthase that we report to be encoded in the terminal end of chromosome D in CLIB89 YALI1, that were previously reported collected in the CLIB122 sequencing project, but not mapped to any chromosomal scaffold [8].

Comparison of genes identified in CLIB89 YALI1 with those reported in CLIB122 YALI0 also identified several that were present in CLIB122 YALI0, but not identified in CLIB89 YALI1. Differences in the mating-type proteins were among these, as expected. In addition, there were multiple differences in transposon sequences of class I (RNA) and class II (DNA) elements further detailed below. In addition, we report 1428 CDS and 88 pseudogenes (Table 4) in CLIB89 YALI1 sequence. The majority of the 1428 CDS were discovered in the SnowyOwl pipeline and lacked transcript or identifiable protein domain support. In addition, compared to the CDS with those properties, a significant fraction of these CDS were relatively short, although longer than 100 codons.

### Transposable elements in CLIB89

*Y*. *lipolytica* displays striking diversity in transposon composition with relics of a Class II (DNA) element and both LTR and non-LTR class I (RNA) elements (Table 5 and S7 Table). Full-length copies of LTR retroelements can undergo recombination between the LTRs resulting in deletion of the internal domain and one LTR copy, generating a so-called solo LTR. Despite fundamental differences in transposition mechanisms, transposable elements (TE) have common distinguishing features. For example, they are typically present in multiple copies per genome. Ultimately both classes are mobilized by transposases/integrases and terminate in a conserved inverted repeat recognized by those enzymes. TG…CA represents the minimal virtually universally conserved terminal inverted repeat, although individual elements exhibit a range of inverted repeat lengths. Insertions are initiated by strand transfer. Because these reactions occur across a DNA helix, reacting positions on the two strands are offset; repair of the single-stranded extensions caused by the offset generates short target-site duplications that flank outside transposon ends [36]. We first searched for Ty1-Copialike and Ty3-Gypsylike conserved reverse transcriptase-coding sequences, and other TE sequences identified in CLIB122 YALI0. However, during identification of allelic tDNAs, we also identified a number of insertion polymorphisms which were characterized by the properties described above that are conserved among solo LTRs. Because we failed to identify a retroelement full-length copy, we designated these as LTRyl7, 8, 9. However, this did not represent an exhaustive search of the CLIB89 genome for novel LTRs.

**Table 5:** Families of transposable elements in CLIB89 and CLIB122.

Class of Transposable Element	CLIB89	CLIB122	Allelic
**Ylli—L1 non LTR retroelement**			
# of Full length (6494 bp)	17	10	7
# of Partial (~3800 bp)	4	2	0
**Ylt1—Ty3/Gypsy retroelement**			
# of Full length (9453 bp)	0	10	n/a
# of Solo LTRs (~715 bp)	0	17	n/a
**Tyl6—Ty3/Gypsy retroelement**			
# of Full length (5.104 kb)	0	1	n/a
# of solo LTRs (276 bp)	0	0	n/a
**Tyl3—Ty3/Gypsy retroelement**			
# of Full length (5973 bp)	1	0	0
# of Solo LTRs (244 bp)	4	4	3
**Mutyl—Mutator-like DNA transposon**			
# of Full length (7413 bp)	0	5	0
# of solo MudrA (3537 bp)	1	1	1
# of solo MudrB (1380 bp)	0	0	n/a
# of solo MudrA like (2628 bp)	1	1	1
# of solo MudrB like (1379 bp)	1	1	1
**LTRyl1 element (Putative)**			
# of Full length	0	0	n/a
# of Solo LTRs (~278 bp)	54	30	30
**LTRyl7 element (Putative)**			
# of Full length	0	0	n/a
# of Solo LTRs (~337 bp)	14	17	14
**LTRyl8 element (Putative)**			
# of Full length	0	0	n/a
# of Solo LTRs (~302 bp)	11	17	9
**LTRyl9element (Putative)**			
# of Full length	0	0	n/a
# of Solo LTRs (~444 bp)	1	5	1

#### DNA Class II elements

After discovery of the DNA transposon Mutator (Mu) in maize [37], Mu and MUtator-Like Elements (MULEs) were found in a number of other species [38]. The fungal MULE, Hop, in *Fusarium oxysporum* is a well-characterized example [39]. Hop has 99-bp perfect terminal inverted repeats and generates a 9-bp target site duplication. Transposition is mediated by a Hop-encoded 836-aa transposase related to the bacterial *mudrA*-encoded MURA protein. The first report of any DNA element in *Saccharomycotina* was the MULE Mutyl discovered in *Y*. *lipolytica* [28]. Mutyl elements have imperfect terminal inverted repeats of 22 bp and are flanked by 9-bp target site duplications. Mutyl encodes a transposase, MudrA, and a second protein of unknown function, MudrB, that is not universally present in non-maize active MuDR elements.

Five Mutyl elements were reported in the CLIB122 YALI0 genome, of which four were full-length and one had an 8-bp deletion. There is evidence for at least one recent Mutyl transposition [9]. In that study, blotting with a MudrB probe failed to identify a complementary sequence in the CLIB89 genome. Given the relatedness of the CLIB122 and CLIB89 strains, this was interpreted to mean that the element might have been acquired by more recent horizontal transmission to the CLIB89 descendant, CLIB122. Consistent with the previous report, MudrB-coding sequence was not found in the CLIB89 YALI1. However, BLAST searches of CLIB89 sequence identified an ORF allelic to CLIB122 YALI0A14971g (YALI1A15017t) the MudrA transposase sequence in the full-length Mutyl element (YALI0A16207) (Table 5 and S7 Table). Thus, partial Mutyl sequence is present in the predecessor strain at the same site as in CLIB122 and were likely shared by vertical transmission. Furthermore, both CLIB89 and CLIB122 both contain isolated ORF sequences that encode proteins similar to MudrA (YALI0A02266g/ YALI1A02682g) and MudrB (YALI0C17193g/ YALI1C24526g). However, because these sequences share little nucleotide similarity with MudrA and MudrB, respectively, these are likely only distantly related to the Mutyl family of transposons.

Fotyl represents a second Class II element that has been identified in *Y*. *lipolytica* (GenBank: CAG33729). This family was discovered as the Fot1 element in *Fusarium oxysporum* [40, 41]. A full-length element of this family was discovered in *Y*. *lipolytica* and dubbed Fotyl. It is a pogo-like element member of the Tc1-Mariner superfamily. A complete copy including terminal inverted repeats was identified on chromosome E and a partial degenerate copy on chromosome A of CLIB122 YALI0 [8]. These are allelic with sequences in CLIB89 YALI1.

#### LTR retrotransposons

LTR retrotransposons populate CLIB89 and CLIB122 genomes (Table 5, S7 Table). These elements are similar to retroviruses in that the upstream LTR contains the promoter for transcription of genomic RNA and the downstream LTR specifies transcription termination and polyadenylation [42, 43]. Retrotransposons typically contain two ORFs. The upstream ORF encodes capsid structural and nucleic acid binding domains and the downstream ORF encodes proteinase, reverse transcriptase and integrase. Elements have devised various mechanisms to ensure an excess of structural proteins over catalytic proteins. These include programmed frameshifting or even splicing to join the respective protein sequences. Two superfamilies of LTR retrotransposons are abundantly represented in eukaryotes: Ty1/Copia and Ty3/Gypsy. However, unlike *S*. *cerevisiae* in which Ty1/Copia elements predominate, no Ty1/Copia elements have been reported in *Y*. *lipolytica*.

At present, two full-length Ty3/Gypsy LTR retrotransposons, Ylt1 [44] and Tyl6 [45] have been described for *Y*. *lipolytica*. Additionally, a partial integrase coding sequence adjacent to an LTR was designated Tyl3, but no full-length sequence has been reported [10, 32, 46]. In the CLIB122 YALI0 assembly, Ylt1 is abundantly represented with 10 full-length and 17 solo LTRs [44] (this study). Tyl6 is represented by one full-length copy and no solo LTRs. Tyl3 is represented by no full-length elements but four solo LTR copies. LTRyl1 is represented by 30 copies of solo LTR sequence [46]. As noted above, CLIB122 was derived from a cross between French strain CLIB89 and American strain CBS6124-2. Amazingly, CLIB89 YALI1 and CLIB89-derived PO1f completely lack both full-length and solo LTR copies of Ylt1 and Tyl6 based on hybridization, draft assembly and complete sequence analysis [19, 44, 45] (this study). However, it has four copies of Tyl3 LTRs three of which are allelic with copies in CLIB122 and 54 copies of LTRyl1 thirty of which are allelic with copies in CLIB122 YALI0. Similar to CLIB122, it lacks full-length versions of the element.

After identification of several novel LTR sequences, we sought to identify novel full-length elements. BLAST analysis of CLIB89 YALI1 sequence used the Ty1/Copia and Ty3/Gypsy conserved reverse transcriptase domains as queries. A truncated *POL* encoding a Ty1-homologous RNaseH was identified adjacent to a copy of LTRyl1. This LTR was not flanked by direct repeats of a target sequence, consistent with it representing the downstream LTR of a truncated element rather than a solo LTR derived from recombination of terminal LTRs. Ty1/Copia and Ty3/Gypsy elements differ in the order of integrase and reverse transcriptase/RNaseH domains. The position of the RNAseH-coding sequence proximal to LTRyl1, the similarity of the sequence to Ty1 RNAseH, and the lack of short direct repeats bordering the LTR argue that the LTRyl1 family is derived from an extinct Ty1/Copia element. This represents the first identification of a Ty1/Copia element in *Y*. *lipolytica*.

#### Full-length Tyl3, a Ty3/Gypsy retrotransposon

**Fig 4:**
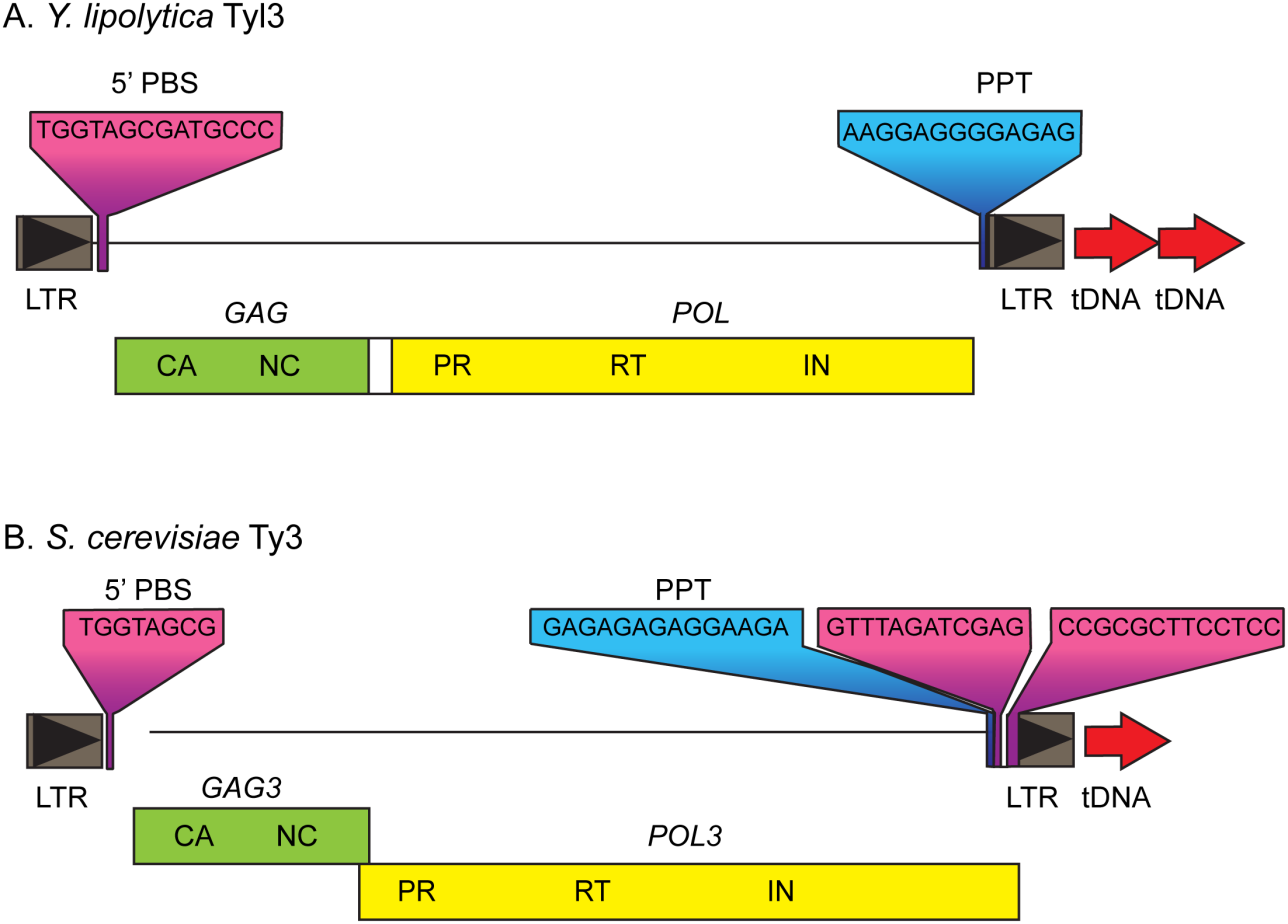
The complete Tyl3 *Y*. *lipolytica* Ty3/Gypsy element. **(A)** Tyl3 was assembled from CLIB89 sequence. Abbreviations are *GAG* (capsid, CA; and nucleocapsid, NC) and *POL* (protease, PR; reverse transcriptase, RT; and integrase, IN), Solid triangles represent LTRs, *GAG* and *POL* are separated by 324 bp. PBS, primer binding sequence complementary to initiator tRNA^Met^ the presumed primer for minus-strand replication; and PPT, polypurine tract the presumed primer for plus-strand replication. The full-length Tyl3 is adjacent to two tDNA sequences. **(B)** Full-length *S*. *cerevisiae* Ty3 is shown for comparison. Features are similarly abbreviated as in 4A.

As described above, tBLASTn analysis was used to search CLIB89 YALI1 coding sequences for those encoding reverse transcriptase, the most highly-conserved retroelement domain. In addition to the Ty1/Copia ORF described above, an ORF with 41% protein identity to the *S*. *cerevisiae* LTR retrotransposon Ty3 reverse transcriptase domain was identified on chromosome C (S3 Text). A tBLASTn search for the conserved core domain of Ty3 integrase showed a predicted sequence with 62% similarity and a BLASTn search showed a 100% match to the previously-reported integrase carboxyl-terminal sequence of Tyl3 reported in CLIB89 (AL414488, AL414575) [45]. Comparison of the candidate LTR of this element and Tyl3 LTR sequences showed 100% identity. Sequences with similarity to nucleocapsid, and the protease active site confirmed the presence of Gag and Pol-like domains. The two ORFs are flanked by the previously described Tyl3 LTR sequences with 6-bp terminal inverted repeats (TGTAAG/CTTACA) (panel A, Fig 4). The outside ends of the LTRs were flanked by 5-bp target site duplications offset by one “T” in the upstream repeat (ATTTTT/ATTTT). Other features of LTR retrotransposons are also present: two nts downstream of the upstream LTR is a sequence complementary to the 3’ terminal 14 nts of initiator tRNA^Met^(CAU), the presumed minus-strand primer. Some patches of initiator tRNA^Met^ complementarity were found in the downstream LTR as well, possibly indicating a bi-partite primer [47]. Just upstream of the 3’ LTR in Tyl3 and corresponding to the position of the polypurine tract (PPT) plus-strand primer for reverse transcription of LTR retroelements is a sequence of 13 consecutive purines. One full-length copy of this element in addition to four solo LTRs were identified in the CLIB89 genome. We conclude that this element constitutes a full-length Tyl3, a fragment of which was previously reported present in CLIB89 based on partial integrase and LTR sequence [10] (Fig 4A).

Unlike the vast majority of Ty3/Gypsy elements found in yeasts that contain a frameshift between the *GAG* and *POL* as illustrated by the prototypic element Ty3 (Fig 4B), the two Tyl3 ORFs, are separated by 324 bp, which includes four stop codons downstream of the *GAG* stop codon Although this is an unusual configuration, it is not without precedent. The Copia-like element *C*. *albicans* Tca2 has a stop codon separating the two ORFs [48], and an internal promoter has been proposed to explain differential expression of structural and catalytic proteins [49]. Some viruses express ORFs from internal ribosomal entry sites [50] and the foamy retrovirus expresses its *POL* functions from a spliced RNA [51]. However, scanning the *Y*. *lipolytica* Tyl3 spacer with software designed to identify viral internal ribosome entry sites [52] and intronic sequences (http://genes.mit.edu/GENSCAN.html)[53] failed to identify either.

Inspection of the Tyl3 allelic site sequence in CLIB122 confirmed absence of Tyl3 sequence or any repeat of the 5 bp sequence duplicated at the ends of the full-length Tyl3 in CLIB89. This result suggests that this empty site in CLIB122 was inherited from the American strain CBS6124-2 or that the transposition in CLIB89 was relatively recent followed by loss of the progenitor full-length element.

Members of the Ty1/Copy and Ty3/Gypsy LTR retrotransposon classes display patterns of targeting by integrase to genomic histone marks (chromodomain class) or RNAP3 genes [42, 54, 55]. A subset of Ty3/Gypsy elements, including the eponymous Ty3, exclusively target RNAP3 transcription start sites [42]. Because tDNA are transcribed into pre-tRNAs which have an approximately 10-nt pre-sequence, the insertion of a 5-bp target site duplication positions retrotransposon sequence 15 to 17 bp upstream of mature tRNA-coding sequence. Sequences flanking Tyl3 and Tyl3 LTRs were analyzed using tRNAscanSE. Analysis showed that Tyl3 is inserted at the likely transcription start site of tDNA^Val^, previously identified in CLIB122 YALI0 sequence. In addition, two of the four Tyl3 solo LTRs are positioned at likely transcription start sites of tDNAs. Two others are less closely related and are inserted 5 bp inside the upstream end of a tDNA and at the 5’ end of a 5S rRNA gene. A full-length copy of Tyl6 was previously identified close to the transcription initiation site of the tRNA^Met^ gene [45]. Comparison of the integrase domains of these elements showed that they are Ty3/Gypsy elements of the class lacking a chromodomain and closely associated with RNAP3 transcription start sites [45, 54, 56] (this study).

#### tDNA-associated LTRs

Analysis of tDNA polymorphisms surfaced three additional sequences with the properties of solo LTR sequences, but for which associated CDS were not identified (Table 5, S7 Table). Based on these observations and in order to avoid conflicting with the nomenclature proposed for extant full-length elements [10] or overlapping with yet-to-be discovered full-length elements for which LTRs have designated numbers, the LTRs discovered in our study were designated LTRyl7, LTRyl8, and LTRyl9. These families grouped by primary sequence and length are characterized by multiple members, and inverted repeat TGT…ACA termini. Most are flanked by short direct repeats of presumed insertion-site sequence.

#### Non-LTR Ylli LINE retrotransposons

LINE retroelements comprise about 40% of the human genome and exist in many other species [57]. They are powerful remodelers of eukaryotic genomes because of their ability to transpose other sequences both in trans and cis. LINES were thought absent from Hemiascomycetes until the discovery of the *Y*. *lipolytica* LINE Ylli in *Y*. *lipolytica* in 2000 [46] and in *Candida albicans* [58]. Ten full-length LINEs were reported in CLIB122 YALI0. In the current study, seventeen full-length elements were identified in CLIB89 YALI1. Much of our understanding of LINES is extrapolated from studies of the active human L1 element [57]. L1 encodes first and second ORFs that are translated into structural and catalytic proteins, respectively. These associate with cytoplasmic RNAs including genomic Ylli RNA and mediate nuclear re-entry, reverse transcription, and subsequent integration. Ylli similar to L1, is a member of the class of LINES in which ORF2 encodes an apurinic-like endonuclease that nicks chromosomal AT-rich sequences. The DNA 3’ end created by nicking primes reverse transcription of the template genomic RNA starting at the downstream end, a process known as target-primed reverse transcription [59]. Reverse transcriptase or repair enzymes complete second-strand synthesis. A distinguishing feature is that reverse transcription is not highly processive so that 5’-truncated LINES tend to accumulate in genomes [57]. LINE insertions terminate in poly(A) tracts diagnostic of their origin as reverse transcribed RNAP2 transcripts and are typically flanked by short direct repeats of target site DNA.

The *Y*. *lipolytica* LINE, Ylli, has generic features of LINEs as well as distinguishing characteristics (Table 5, S7 Table). Ylli encodes proteins of 714 and 1,300 aa, the second of which contains homology to reverse transcriptase and apurinic-type endonucleases. It exists in multiple 5’ truncated copies and is associated with downstream poly(A) tracts [11]. However, Ylli is distinct from other LINEs in that target-site duplications have not been identified. We speculate that target-site duplications exist, but are too short to be identified, or as sometimes is the case, the ends of the element have non-templated reverse transcribed nucleotides which confound identification of the ends of insertions. Underscoring differences between the CLIB89 and CLIB122 genomes, as mentioned above, there are seventeen apparently full-length, 6.5-kb Ylli insertions in CLIB89 YALI1 and ten in CLIB122 YALI0. In addition, in CLIB89 YALI1, there are seven Ylli sequences greater than 1.0 kb and one hundred and four fragments between 30 bp and 1 kb in length (Table 5 and S7 Table).

An intriguing feature of metazoan genomes is the expansion of short sequences related to RNAP3 genes referred to as *S*hort *IN*terspersed *E*lements (SINEs) typically about 300 bp in length. Work in human cells has demonstrated that LINEs retrotranspose these RNAs [60]. SINEs are characterized by internal RNAP3 promoter elements in the first segment of the sequence and associated LINE or other RNAP3 sequences in the second half of the element [61]. Mobilized SINEs are typically associated with downstream poly(A) tracts [62]. A particularly unique aspect of the *Y*. *lipolytica* genome is the expansion of tDNAs and dimeric RNAP3 genes relative to other Ascomycetes. This poses an intriguing parallel with the origin of SINEs as dimeric RNAP3 genes. We speculate that retroelement LTRs associated with RNAP3 genes could have provided poly(A) transcript templates for LINE-mediated proliferation of tDNAs in *Y*. *lipolytica*.

#### Sources of transposable element differences between CLIB89 and CLIB122

Transposable elements provide for much of the variation within species and are even proposed to account for aspects of speciation. Although CLIB89 was one of the parental strains used to derive CLIB122, we observed striking differences in TE composition. It was of interest to estimate the extent to which segregation versus active transposition contributed to these differences. The abundant Ylt1 in one strain but none in the other strain was particularly striking. We considered three possible hypotheses for the differences between these two closely-related strains in Ylt1 and other elements: 1) there are similar positions and numbers of the element of itnerest in both strains, but ancient versions in the CLIB89 lineage have degenerated and are no longer readily detectable by BLAST analysis; 2) copies were possibly more abundant in CBS6124-2 or CLIB89 and simply segregated differentially; and 3) a wave of retrotransposition sometime after the cross of the *Y*. *lipolytica* strains resulted in differential proliferation of elements between the two strains.

These hypotheses make distinct predictions regarding the degree of variation in sequence flanking allelic and non-allelic TE. Hypothesis one predicts that elements present in CLIB122 and as relics not identified by BLAST analysis in CLIB89 could be identified by reconstructing the sequence in CLIB122 without the insertion and aligning it to that region in CLIB89. Hypothesis two predicts that elements existing in CLIB122, but not CLIB89, such as Ylt1, would be embedded in sequence inherited from CBS6124-2 and so would be relatively enriched in variants when compared to the same region from CLIB89. Hypothesis three predicts that although some CLIB122 elements might be flanked by variants, if overall an element transposed after the CLIB89 X CBS6124-2 cross, then insertions sites would be randomly distributed across DNA from each parent and *overall* mismatch density would be similar to average genomewide mismatch densities.

To distinguish among these possibilities we took Ylt1 as an example of an element over-represented in CLIB122 and Tyl1 as an example of an element over-represented in CLIB89. We first manually reconstructed 27 full-length element and LTR insertion sites in CLIB122 to derive “naïve” sequences for comparison to CLIB89. BLAST analysis showed that they these empty sites existed in CLIB89, thereby excluding the interpretation that ancient relics in CLIB89 represented insertions identified solely in CLIB122.

**Fig 5:**
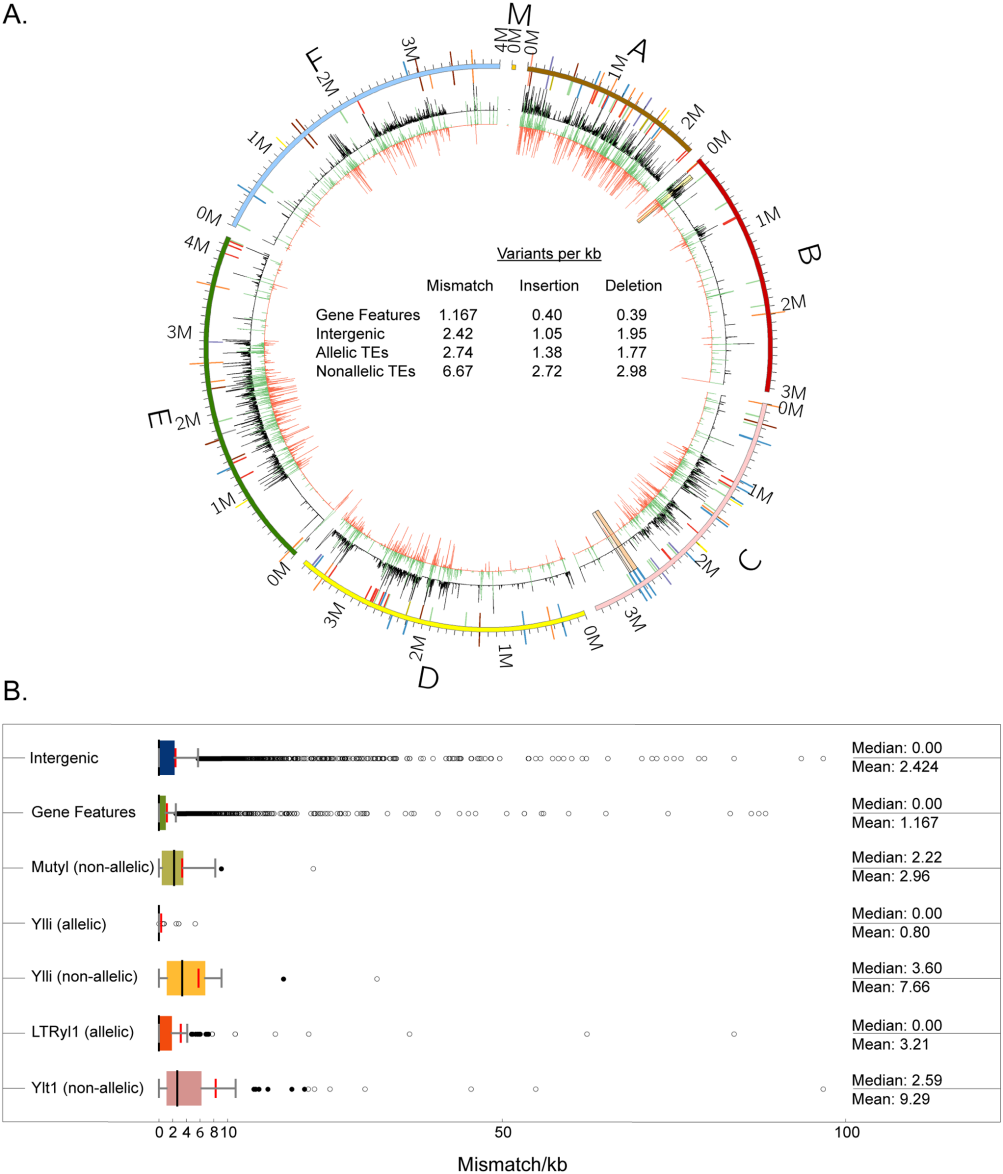
Genomic differences between CLIB89 YALI0 and CLIB122 YALI1 assemblies. (A) Circos diagram illustrating the sequence variation between CLIB122 YALI0 and CLIB89 YALI1 and the locations of annotated TE. Outer circle: TE in both assemblies are represented by colored bars projected outward (CLIB89 YALI1) or inward (CLIB122 YALI0) from the chromosome ideograms: Ylli (blue), Mutyl (purple), Ylt1 (red), LTRyl1 (green), LTRylt7 (orange), LTRyl8 (maroon), LTRyl9 (gold), Tyl3 (yellow), Tyl6 (grey). Inner circles: The black track indicates the mismatch density between corresponding regions between CLIB89 and CLIB122 in each chromosome. The green and orange tracks represent insertions and deletions respectively relative to the CLIB122 YALI0 reference genome assembly. (B) Mismatch density of TE and flanking iLoci of allelic compared to nonallelic elements. The mismatch density (mismatch/kb) of each individual element and flanking iloci is shown. Mean and median (red and black horizontal bars, respectively); near and far outliers (black and white circles, respectively).

In order to more quantitatively evaluate the second and third hypotheses, variant densities (M = mismatch/kb, I = insertions/kb, D = deletions/kb) were quantified and averaged for the four iLoci surrounding the TE insertions (two sequential iLoci per flank) to estimate the extent of differences in the region of the TE. Results of this analysis showed that as expected overall intergenic iLoci averaged higher variant density (M = 2.42, I = 1.05, D = 1.95) than gene feature iLoci (M = 1.17, I = 0.40, D = 0.39)(Panels A and B, Fig 5; S8 Table). In contrast, the nonallelic family Ylt1with 10 full length and 17 solo LTR members exhibits flanking regions of even greater variant density (M = 9.29, I = 2.25 nd D = 3.09) whereas the 30 LTRyl1 allelic elements of the 54 LTRyl1 total elements in CLIB89 YALI1 are embedded in regions of lower variant density (M = 3.21, I = 1.54, and D = 2.52) in contrast to LTRyl1 non-allelic members (M = 4.65, I = 2.68, and D = 1.93) and Ylt1 non-allelic elements. The LINE Ylli showed a similar pattern (non-allelic, M = 7.66 versus allelic M = 0.80). Furthermore, overall, non-allelic members of TE families occurred in regions of greater variant density (M = 6.67, I = 2.72, and D = 2.98) compared to allelic members (M = 2.74, I = 1.38, D = 1.77)(Panel A, Fig 5 Circos; Panel B, Fig 5 box plots; S8 Table). The observed differences in the mismatch/kb and insertion densities in comparing all allelic TE to non-allelic TE are significant (p = 0.0027 and p = 0.01, respectively), but the deletion density difference is less (p = 0.082). Overall, therefore while we cannot formally exclude the contribution of TE mobilization subsequent to the CBS6124-2 X CLIB89 cross as an explanation for gross differences between progeny strain CLIB122 and parent CLIB89, data are consistent with hypothesis 2, namely that non-allelic families such as Ylt1 CLIB122 elements were plausibly inherited together with flanking sequence from the CBS6124 parent, rather than supporting hypothesis 3, that subsequent to generation of CLIB122, new insertions occurred and were randomly distributed into intergenic sequence. The analysis developed for this study provides a new tool for differentiating the impact of transposition and chromosomal inheritance to phylogenetic analysis of closely-related strains.

## Conclusions

*Y*. *lipolytica* is being rapidly adopted as a mainstream species for single cell, biosustainable production of chemicals. In order to provide an improved basis for manipulation of strains related to industrial strain CLIB89, we used hybrid, next-generation sequencing coupled with Irys long-range genome mapping to assemble the CLIB89 genome, making it the first *Y*. *lipolytica* genome in which single contigs comprise each chromosome. One copy of the rDNA was completely sequenced and tandem repeats of rDNA were documented in the terminal regions of four chromosomes.

Intriguing aspects of the *Y*. *lipolytica* biology are the greater size of the genome and abundance of RNAP3 species compared to other fungal genomes. We speculate that although identified retrotransposons comprise a relatively small portion of the *Y*. *lipolytica* genome, they have not only contributed to its expansion but have mediated its adaptation to the diverse environments in which it is found. This possibility is supported by several observations. First, *Y*. *lipolytica* is striking for the diversity of its elements. These include previously reported DNA element Mutyl, multiple LTR elements and LINE Ylli in full and partial copies. Second, the apparent abundance of LINE fragments and solo LTR-like sequences is consistent with occupation by many now-extinct retrotransposons. Third, autonomous LINES and LTR retrotransposons are present and are known to mediate mobilization of other genomic sequences, including RNAP3 transcripts enabled to evolve into SINEs. We speculate that the surprisingly extensive differences in retrotransposons between related *Y*. *lipolytica* strains CLIB89 and CLIB122 relate in large part to differences between the parental CBS6124-2 and CLIB89 genomes, and might contribute to inefficient mating between these strains despite mating-compatible mating types.

The current findings further underscore the usefulness of a second, independent *Y*. *lipolytica* genome sequence. Assembly of a six-contig genome for *Y*. *lipolytica* will empower ongoing molecular manipulation of this important and novel organism.

## Materials and Methods

### Strains and culture conditions

#### CLIB89 and CLIB122 strain relationship

*Y*. *lipolytica* strains in industrial use are descended from strains isolated from diverse ecological niches and in some cases, crossed with other isolates and sporulated to yield new haploid strains. One strain with significant genomic sequence available is W29, which was isolated from waste water in Paris, France. It is designated in the Collection de Levures d’Interet Biotechnologique (CLIB) as CLIB89. A second is E150, which is a haploid derivative of a cross of CBS6124-2 isolated from an American corn processing plant and CLIB89. This strain was designated CLIB122. The CLIB122 genomic sequence is the most thoroughly characterized *Y*. *lipolytica* genomic sequence (http://www.ncbi.nlm.nih.gov/genome/genomes/194?). In order to have a comparably detailed assembly of the progenitor strain CLIB89, haploid *Y*. *lipolytica* CLIB89 was purchased from ATCC (ATCC 20460) and the genome sequence was determined.

#### Culture conditions

For genomic sequencing, CLIB89 was cultured in 2% Yeast extract-1% Peptone 2%-Dextrose (YPD) [63]. For Illumina sequencing DNA was extracted according to standard protocols; RNA was removed by RNase digestion [64]. DNA was sheared to appropriate length using the S2 Ultrasonicator (Covaris). Libraries were prepared for sequencing using the NEXTflex Rapid DNA-Seq Kit (Bioo Scientific). For PacBio sequencing, cells were spheroplasted by treatment with Zymolyase (Seikagaku Corporation). Spheroplasts were pelleted, lysed and digested with RNaseA (Fermentas) and Proteinase K (Fisher Scientific). DNA was isolated and then eluted from the Qiagen Anion-Exchange Resin. For datasets YLP13 and YLP14, corresponding to PacBio RS sequencing, high MW DNA was extracted with the Genomic-Tip 20/G kit (Qiagen). DNA was fractionated to 4–50 kb using a BluePippin pulsed-field gel electrophoresis system (Sage Sciences). PacBio SMRT Bell sequencing libraries were prepared using the manufacturer’s DNA SMRT kit. Results of RNA sequencing will be published elsewhere. Transcripts from cells grown under several conditions were combined in order to maximize the potential of transcriptomics to identify reading frames. For RNA sequencing cells were lysed, and RNA was processed into KAPA stranded libraries for Illumina PE100 sequencing according to manufacturer’s instructions.

### DNA-Seq

#### Next-generation Illumina and PacBio sequencing

For the genome assembly described below (Implementation), four DNA sequencing datasets were generated from a combination of Illumina HiSeq 2500 and PacBio RS II reads (Table 1). Datasets YL97B and YL110, corresponded to Illumina single-read (SR) 97 cycles and paired-end (PE) rapid run 110 cycles, respectively. For YLP13 and YLP14 sequencing was conducted on eight and four SMRT cells, respectively. Longer inserts were prepared for YLP14 (Table 1). Illumina sequencing data were processed and demultiplexed using CASAVA1.8.2. PacBio RS II was processed using SMRT Portal.

#### *De novo* genome assembly

High-quality short Illumina reads and long PacBio RS II reads were combined in a custom hybrid approach to assemble the CLIB89 genome (Table 1). Details are provided in S1 Text.

#### Contig assembly of Illumina short reads

Sequences from YL97B and YL110 Illumina datasets were assembled into contigs and filtered as described in S1 Text, Materials and Methods using the Velvet *de novo* assembler [65].

#### Extending and scaffolding of Illumina contigs with long PacBio reads

PacBio long reads from datasets YLP13 and YLP14 were used to scaffold the *de novo* contigs generated from Illumina analysis. This PacBio scaffolding allowed the determination of both immediate mate(s) to each contig, as well as long distance information up to four mates away. This information was used to determine the placement of contigs spanning repetitive regions in the genome. The PacBio scaffolding was accomplished through the following four steps: i) matching PacBio long reads with the first-stage contigs via BLAST [66] and extending each BLAST hit via a combination of in-house software and ClustalW [67] to generate mapped sequences with computed percent identity; ii) filtering to remove unreliable mapped sequences, sequences that will not provide scaffolding information, and sequences leading to ambiguous or conflicting cases; iii) selecting the closest, non-repetitive right-mate contig and incorporating that into the growing assembly; and iv) identifying the correct mate for a repeated contig via long-distance contig information.

#### Assembling junctions between extended contigs

Successful scaffolding of contigs from the previous stage enabled the identification of junction sequences. Moreover, the consensus junction sequences resulting from the multiple sequence alignment was estimated to have an error rate of 8%, significantly lower than the error rate of a single PacBio read.

The junction sequences were obtained via ClustalW by generating a multiple sequence alignment (MSA) of PacBio reads from datasets YLP13 and YLP14 spanning each junction region plus 200 bp into each flanking de novo contig. Positions in the MSA were selected for the consensus sequence only when: i) enough reads confirmed its existence, and ii) the reads had high agreement, where the two parameters values (i, ii) were optimized for each junction for which the target consensus sequence was known. These junction sequences were further corrected for consensus by utilizing previously unused reads from datasets YL97B and YL110, where unaligned single reads in YL97B with respect to the contigs were reused by Velvet to generate additional comparison contigs, and paired reads in YL110 with part of a read in the junction regions were extracted with Eland v2e. The new contigs and selected paired reads were used iteratively to complete the error-correction. The resulting scaffolds were assigned chromosomal names based on comparison to chromosome designations in the CLIB122 assembly.

#### Bridging the gap in chromosome C and extending chromosome ends

From the aforementioned assembly pipeline, two scaffolds matched with chromosome YALI0C of the CLIB122 assembly. The junction sequence was assembled by: i) aligning paired-reads in YL110 iteratively to the scaffold ends to extend the scaffold sequences; ii) extending contigs by aligning unused PacBio reads at each iteration of scaffold extension via the same protocol for PacBio reads described in the previous two stages; iii) stopping the iterations when enough PacBio reads were matched to both scaffold ends. The same approach was used to extend the chromosome ends until no more bases could be added. The chromosome C contig joint was confirmed to overlap by PCR utilizing primers annealed to unique sequences at the ends of the two contigs (S1 Table).

#### Mapping of chromosomal ends

Of the twelve chromosomal ends, two were similar between the CLIB89 and CLIB122 assemblies; five were longer in CLIB122. Because these five CLIB89 sequences overlapped they were extended into the CLIB122 assembly (S1 Text).

In an effort to extend the chromosomes as close to the telomeres as possible, five CLIB89 terminal sequences were extended by joining termini of CLIB89 contigs to overlapping CLIB122 sequences and these joints were verified by existing overlapping long reads. CLIB122 sequence was appended to the ends of the CLIB89 assembly as follows: YALI0B 3' end position 3,044,622 (387 bp); YALI0C 5' end 1 8,912 bp; YALI0C 3' end position 3,353,699 (12,578 bp); YALI0D 5' end 1 3,177 bp; YALI0E 3' end position 4,188,128 bp (10,433 bp); YALI0F 3' end position 3,999,287 bp (3,679 bp). PCR amplification utilizing primers 4956/4957, 4958/4959, and 4960/4961 were used to confirm these additions to the 5’ end of YALI1C, the 3’ end of YALI1B, and the 5’ end of YALI 1D respectively. A flanking primer in the CLIB122 sequence together with a rDNA primer was used to confirm the position of one rDNA repeat. Because the *Y*. *lipolytica* rDNA genes occur as tandem repeats in multiple clusters, a complete rDNA sequence and non-transcribed spacer could be generated using outward priming oligonucleotides based on a single fragment of rDNA sequence in CLIB122 (S3 Table). This demonstrated the occurrence of rDNA sequence on chromosome F. However, there are multiple clusters of rDNA reported in *Y*. *lipolytica* [8] and it is therefore not possible to determine that the intergenic sequence amplified by PCR was derived from chromosome F.

#### Irys long-range mapping analysis

Irys long-range genome mapping (BioNano Genomics, Inc.) enables comparison of chromosomal restriction maps to chromosomal sequence for genome sequence validation and genome comparisons. In our application DNA molecules of average length 285 kb were subjected to nicking with a single-strand-specific restriction endonuclease followed by nick translation to introduce fluorescent tags. DNA was stained and imaged during low-voltage electrophoresis in Irys instrument nanochannels.

The raw image data were converted to digital representations of the restriction site-specific labeling and the resulting tag patterns of 621,169 molecules were assembled *de novo* into 31 contigs using IrysSolve software (Fig 1A, Table 3). These contig tag patterns were aligned to a virtual restriction tag pattern generated from the hybrid Illumina-PacBio YALI1 sequence. This alignment showed overall agreement between the two assemblies. However, the length of the Irys assembly was 25.246 Mb, significantly longer than either CLIB89 or CLIB122 sequence assemblies. This difference could be explained by heterogeneity of the lengths of chromosomal ends in which Irys mapping was biased for the longest extensions or redundancy in mapping terminal repeated regions. Consistent with these possibilities, inspection of the Irys assembly in telomeric regions showed that Irys contigs at the left end of chromosomes A, C, and F and the right end of chromosome B were each nearly 1 Mb longer than the corresponding YALI1 chromosomes and contained a distinctive ~10 kb unit repeat. Analysis of these regions is discussed further below.

DNA extraction and labeling was according to BioNano Genomics protocols. Briefly, CLIB89 cells were spheroplasted by treatment with Zymolyase (Seikagaku Corporation), immobilized in low-melting point agarose matrix, and treated with proteinase K (Qiagen), washed with TE, digested with RNAse and washed again with TE. Agarose was melted and digested with GELase (Epicentre Biotechnologies) to recover genomic DNA. Buffers were exchanged by drop dialysis and DNA was stained according to QuBit dsDNA HS kit instructions, sonicated and quantified on a Qubit Fluorometer. DNA molecules of average length 285 kb were subjected to digestion to completion with single-strand nickase Nt.BspQI. Finally 300 ng of DNA was labeled by limited-drive nick translation in the presence of a fluorophore-labeled nucleotide. Labeled nicks were repaired using a thermostable polymerase and ligase.

Eight microliters of DNA at a concentration of 4.6 ng/microliter DNA was loaded into a flowcell of the IrysChip for imaging in the Irys instrument in a low voltage electric field controlling sample flow of individual molecules through nanochannels for 30 cycles. Under laser excitation images of DNA were captured by EM-CCD in the Irys Instrument and using AutoDetect software processed into digitized molecule image files. IrysView software was used to analyze and visualize these data. IrysSolve running on an independent server was used to assemble the *Y*. *lipolytica* CLIB89 genome. This analysis generated 31 contigs. These contigs were aligned with YALI1 sequence at a confidence threshold of negative log of P value = 45. Chromosomes A, B, C, and F showed substantial extensions relative to the hybrid sequence assembly. The extended regions displayed striking tandem unit repeats of ~10kb tagged at intervals consistent with the Nt.BspQI digest pattern predicted for rDNA-coding sequence (S4 Table).

### Genome annotation

#### Gene identification using RefSeq/NCBI *Y*. *lipolytica*

CLIB122 gene sequences available in RefSeq Yl were compared to the CLIB89 assembly via a combination of various BLAST [66] and Exonerate [23] alignment algorithms to maximize consistency with previously identified genes. To find the equivalent features across the assemblies, Exonerate was used to map CLIB122 RefSeq YI features by their respective nucleotide sequences, both with and without flanking nucleotide sequences, to the CLIB89 assembly. The Exonerate mapping with flanking sequences identified the equivalent genes in the two assemblies, while mapping without flanking sequences helped to identify additional paralogs present in CLIB89. The EST2GENOME alignment algorithm was used on protein-coding sequences to take into account intron variation, while AFFINE:LOCAL (a local alignment similar to the Smith-Waterman-Gotoh algorithm) was used on non-coding sequences. Loci mapped in CLIB89 were assigned corresponding RefSeq Yl annotations.

#### Gene identification using YGAP

YGAP is an automated yeast/fungal genome annotation services available online at http://wolfe.ucd.ie/annotation/. CLIB89 and CLIB122 assemblies were analyzed in parallel with the seven chromosome sequences of each assembly used as “Scaffolds” in creating a new YGAP project. In addition to identification of coding sequences, YGAP identifies tRNA genes (tDNAs) using tRNAscan-SE [68] and excludes them from coding regions, and identifies Ty LTR retrotransposons. For a parallel comparison, CLIB122 YALI0 was analyzed using YGAP as well. The YGAP CLIB89 gene set included 6,448 loci, 5,938 protein-coding sequences, and 510 tDNA (chromosomal and mitochondrial), compared to YGAP CLIB122 gene set of 6,467 loci with 5,930 protein-coding sequences, and 537 tDNA (including mitochondria and chromosomal).

#### Gene refinement and validation using SnowyOwl HMM

The SnowyOwl pipeline is based on ranking models generated by various HMM gene predictors [22]. To run the SnowyOwl pipeline for the present study, RNA-Seq reads were mapped and assembled into *de novo* transcripts via TopHat and Cufflinks [69], which was then used as the initial transcript model to help train the subsequent SnowyOwl pipeline. Cufflinks-assembled transcripts were aligned to the CLIB89 assembly using Tophat2, and then used to generate initial models for training a Hidden Markov Model gene predictor, together with the intron and transcribed positions revealed by the Tophat2 mappings. Next, Genemark-ES [70, 71] was run on the CLIB89 assembly to generate GeneMark gene models, which were ranked based on RNA-seq data to produce a set of high-confidence gene models as the training set for another *ab initio* gene predictor, AUGUSTUS [72]. AUGUSTUS was run several times with different parameter settings and generated models scored using homology-based evidence, exon-intron boundaries and coverage. Highly-scored models with the best agreement with RNA-Seq data and homology evidence were preserved by default; imperfect models that lacked similarly high RNA-Seq coverage were also captured and added to the list, but were flagged as imperfect. The homology evidence used included BLASTx sequence homology search against the NCBI fungal databases and Uniprot protein database [73]. In total, from the HMM models, SnowyOwl predicted 7,482 protein-coding sequences over 100 codons in length on the CLIB89 assembly—5,464 of which corresponded to RNASeq transcripts. Of the 7,482 SnowyOwl protein-coding sequences, 6,311 corresponded to protein-coding sequences identified by either YGAP or RefSeqYl, leaving a total of 1,171 additional protein-coding sequences identified uniquely by SnowyOwl (Fig 2).

#### Final merged set of annotated genes

Existing locus/gene names from CLIB122 YALI0 were reassigned as locus/gene names in the independent CLIB89 YALI1 assembly (S2 Table). UniProt IDs present in the RefSeq Yl were retained in the annotations. The RefSeq mapped sequences, YGAP genes, and SnowyOwl protein coding sequences used common coordinates based on the CLIB89 DNA-seq assembly. The output files of the three pipelines were merged together via the LocusPocus locus-based annotation script in the Aegean Toolkit [74] (http://standage.github.io/AEGeAn). Further processing based on coding sequence length and integrity were performed using in-house scripts that then generates both IGV ready gff3 file and the TBL file for NCBI submission. The TBL undergoes quality checks through NCBI’s Sequin annotation upload service (http://www.ncbi.nlm.nih.gov/projects/Sequin/download/seq_download.html), where problematic gene features are resolved through iterative manual curation.

#### Consensus defined solo LTR identification

Genome sequences for *Y*. *lipolytica* CLIB89 in FASTA format were obtained from hybrid assembly of Illumina and PacBio reads. For analysis of CLIB122 Genolevures database sequences were used [(previously) http://www.genolevures.org/index.html#; (currently) Genome Resources for Yeast Chromosomes website (http://gryc.inra.fr) and http://www.ncbi.nlm.nih.gov/genome/genomes/194?].

#### Allelic genomic features and variants analysis

Genome features were extracted from both CLIB122 YALI0 and CLIB89 YALI1 assemblies. For both lists of genome features, we used the locuspocus program of the AEGeAn toolkit [75] to designate both annotated genomic features and intergenic regions as separate interval loci (iLoci). A two-layered Exonerate alignment (first by using CLIB122 iLoci as query and CLIB89 iLoci as targets and second by vice versa) was used to determine allelic iLoci between the two assemblies, as well as to quantify the number of mismatches and indels between the allelic iLoci (S8 Table). In our analysis, nucleotides present in CLIB89 and not CLIB122 was classified as an insertion and vice versa. Variants density is reported as number of Mismatches/insertion/deletions per kb.

Transposable elements of CLIB89 YALI1 (reported in S7 and S8 Tables) were mapped to the CIRCOS diagram according to their genomic coordinates. To map the transposable elements of CLIB122 YALI0, two methods were utilized. CLIB122 Elements that are allelic were mapped to the same chromosomal location as the CLIB89 counterpart. For CLIB122 elements that were not allelic, flanking iLoci were then utilized to assign a corresponding genomic location.

#### Mitochondrial genome analysis

The mitochondrial genome of Y. lipolytica was previously annotated and reported [76, 77]. Mitochondrial genome analysis was performed utilizing RNAweasel (http://megasun.bch.umontreal.ca/RNAweasel/)[78], an automated annotation program for organellar genomes with intron detection and other features tuned for mitochondrial genomes with manual adjustments. Variants analysis with CLIB122 mitochondrial assembly was performed using the SNP detection pipeline of MUMmer (http://mummer.sourceforge.net/) by using CLIB122 as query and CLIB89 as target to be consistent with the chromosomal variants analysis.

## Supporting Information

S1 Fig Dot matrix comparison of CLIB89 YALI1 and CLIB122 YALI0 genomes. Genomes were globally compared to themselves and to each other by alignment of assemblies using a dot matrix program, MUMmer [79]. Red shows agreement in forward sense and blue in reverse sense. Chromosomes are compared in alphabetical order A to F and mitochondrial genome, M. Chromosome B, bottom panel shows expanded view of 51-kb inversion. Chromosome C, bottom panel shows expanded view of 54-kb repeat region.
(PDF)

S2 Fig PCR confirmation of the reported genomic inversion on chromosome B. Primer pairs JY5118/JY5119 and JY5120/5121 were predicted to generate a PCR product in the CLIB89 YALI1 assembly. Lanes 1–4 utilized CLIB89 genomic DNA as template. Lanes 5–8 used PO1f genomic DNA as template. The primers used in the PCR reactions were detailed as follows: lane 1 and 5 (JY5118/JY5119), lanes 2 and 6 (JY5120/JY5121), lanes 3 and 7 (JY5118/JY5121), lanes 4 and 8 (JY5119/JY5120).
(TIF)

S1 Table Primers. (XLSX)

S2 Table CLIB89 YALI1 genomic and mitochondrial coding sequences mapping to CLIB122 YALI0 and annotations. (XLSX)

S3 Table CLIB89 All pipeline merged features. (GFF3)

S4 Table Optimized features. (GFF3)

S5 Table Genes unique to CLIB89 YALI1. (DOCX)

S6 Table YALI1 RNAP3-transcribed genes. (XLSX)

S7 Table CLIB89 retroelements Ylt1, Tyl1, Tyl3, Tyl6, LTRyl7, 8, and 9, and Ylli. (XLSX)

S8 Table Global comparisons of CLIB89 YALI1 and CLIB122 YALI0 genomes. (XLSX)

S1 Text Supplemental materials and methods. (DOCX)

S2 Text RDNA sequence. (DOCX)

S3 Text Tyl3 sequence. (DOCX)

S4 Text LTRyl7, 8, and 9 consensus sequences. (FA)
