# Supplementary material for: Sequence Assembly of Yarrowia lipolytica Strain W29/CLIB89 Shows Transposable Element Diversity
Source: PLoS One. 2016 Sep 7;11(9):e0162363. doi: 10.1371/journal.pone.0162363 (PMC5014426; doi:10.1371/journal.pone.0162363)

YALI0A – YALI1A Mummer Dot Matrix Comparison

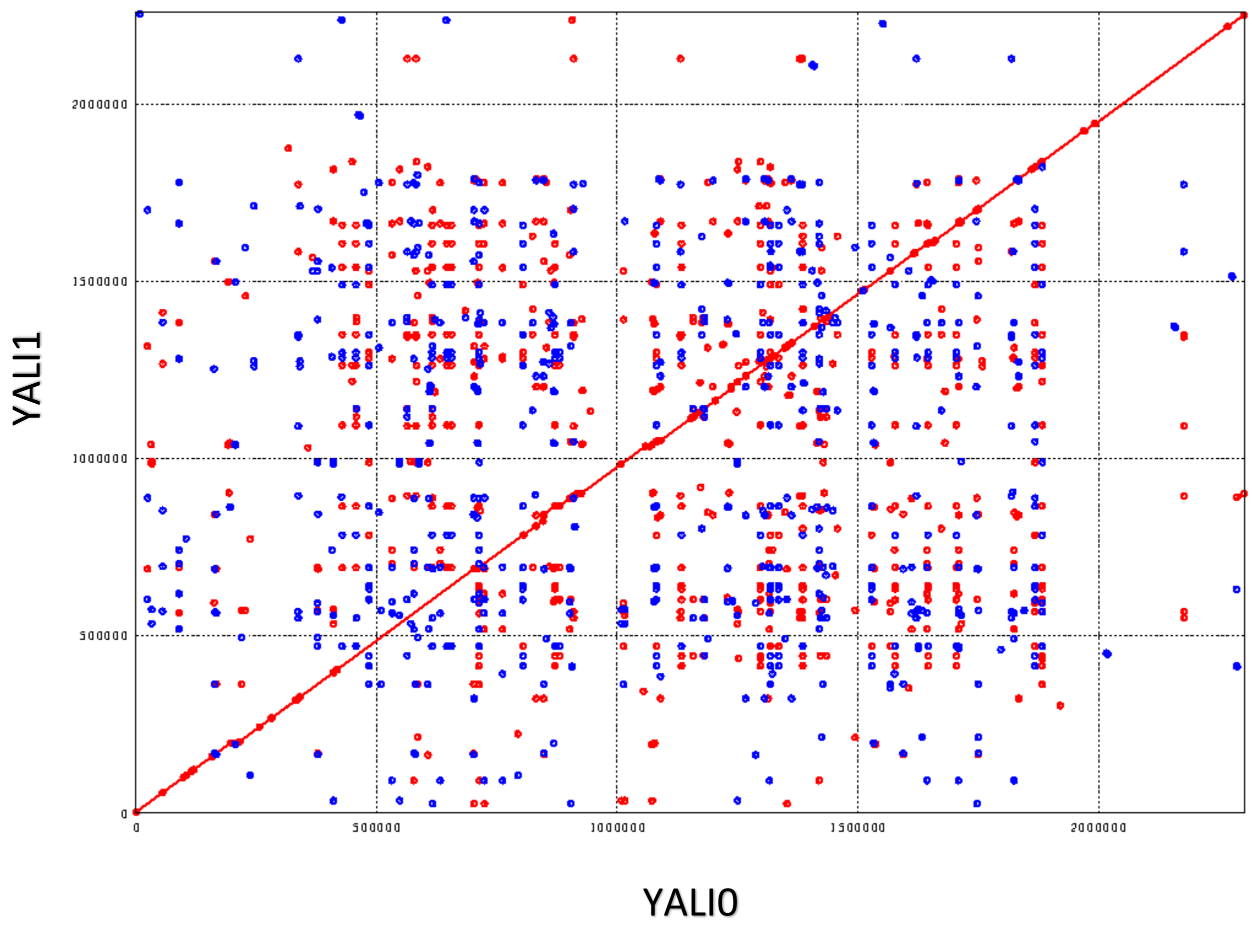

YALI0B – YALI1B Mummer Dot Matrix Comparison

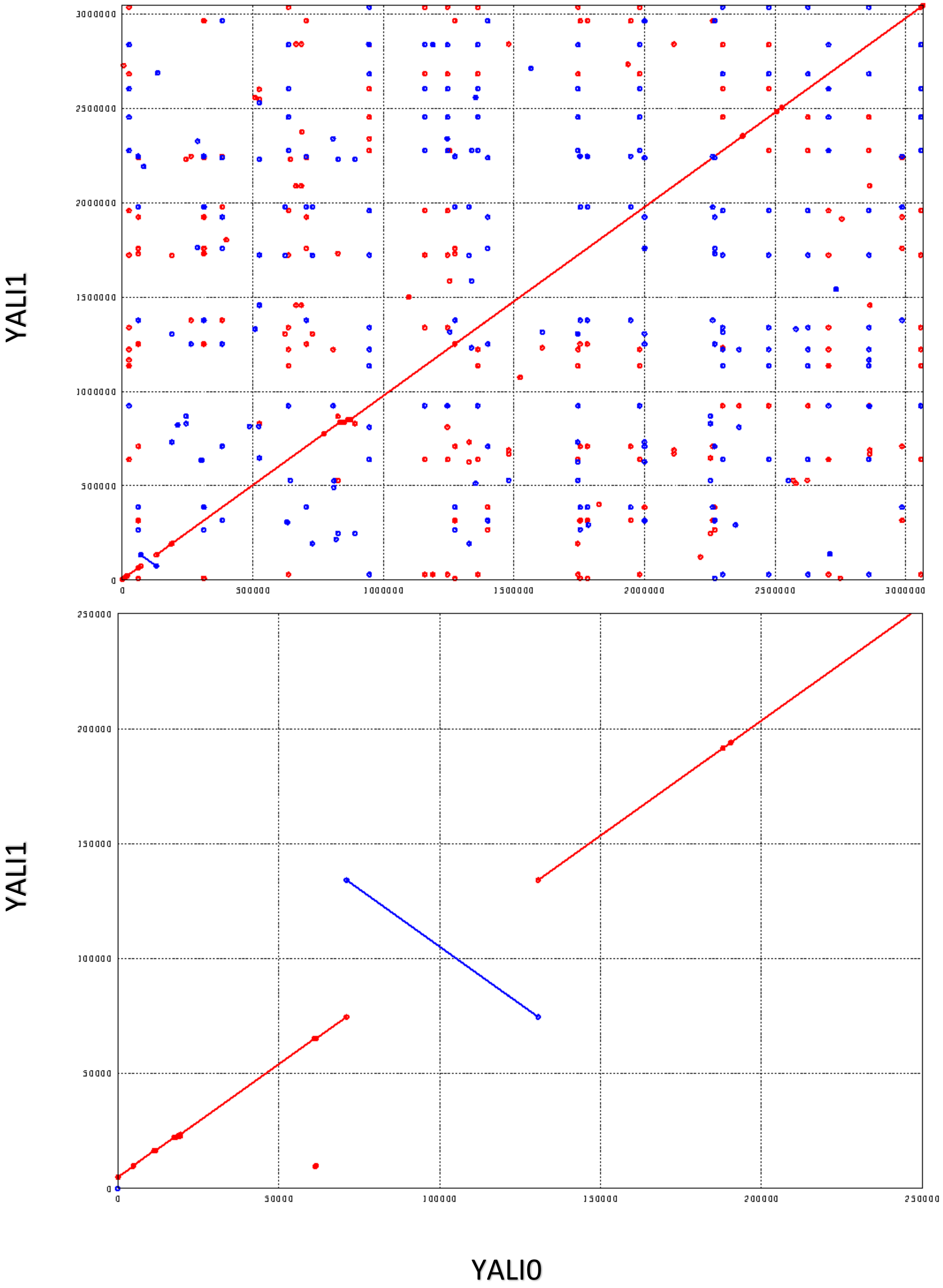

YALI0C – YALI1C Mummer Dot Matrix Comparison

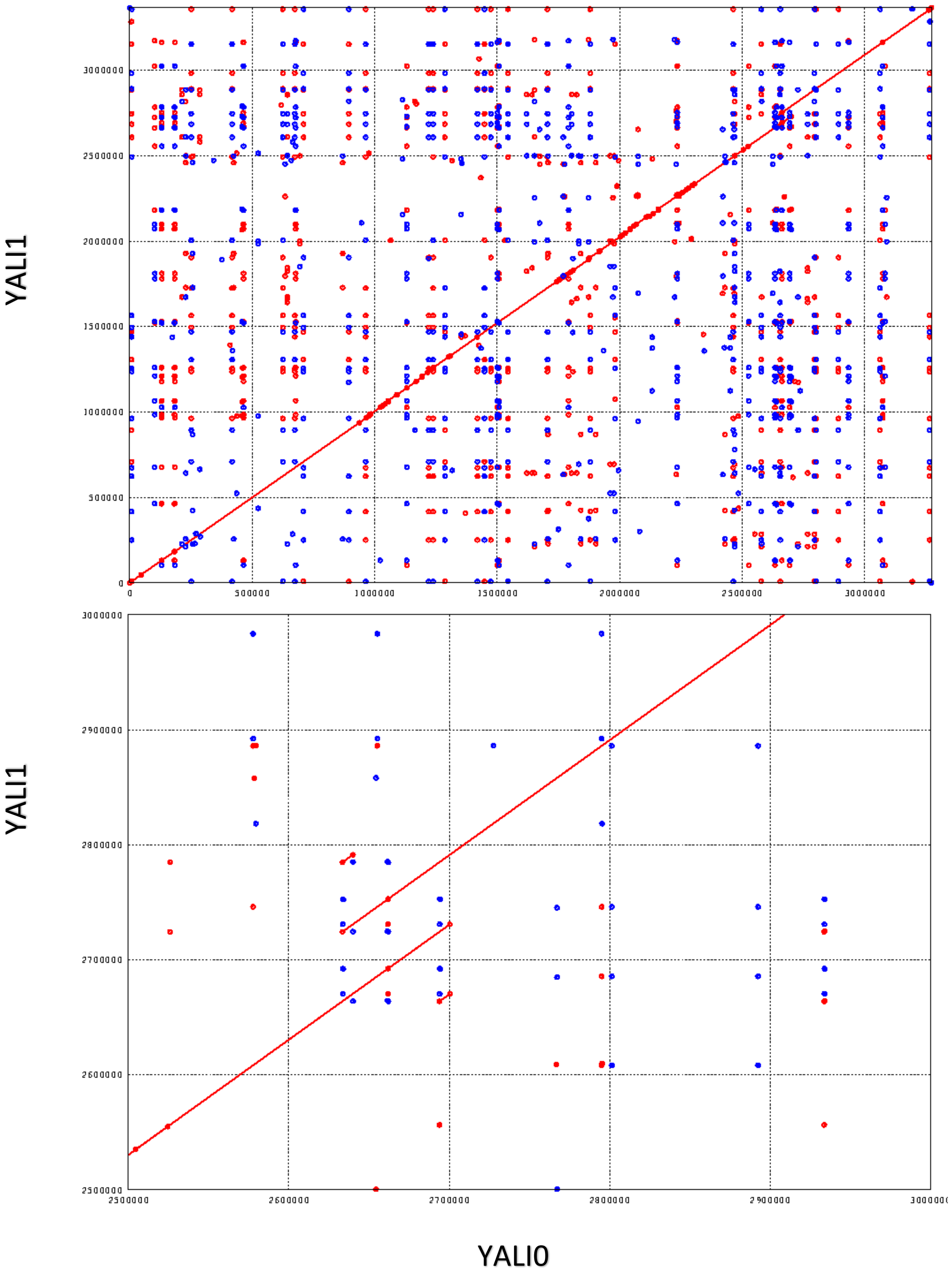

YALI0D – YALI1D Mummer Dot Matrix Comparison

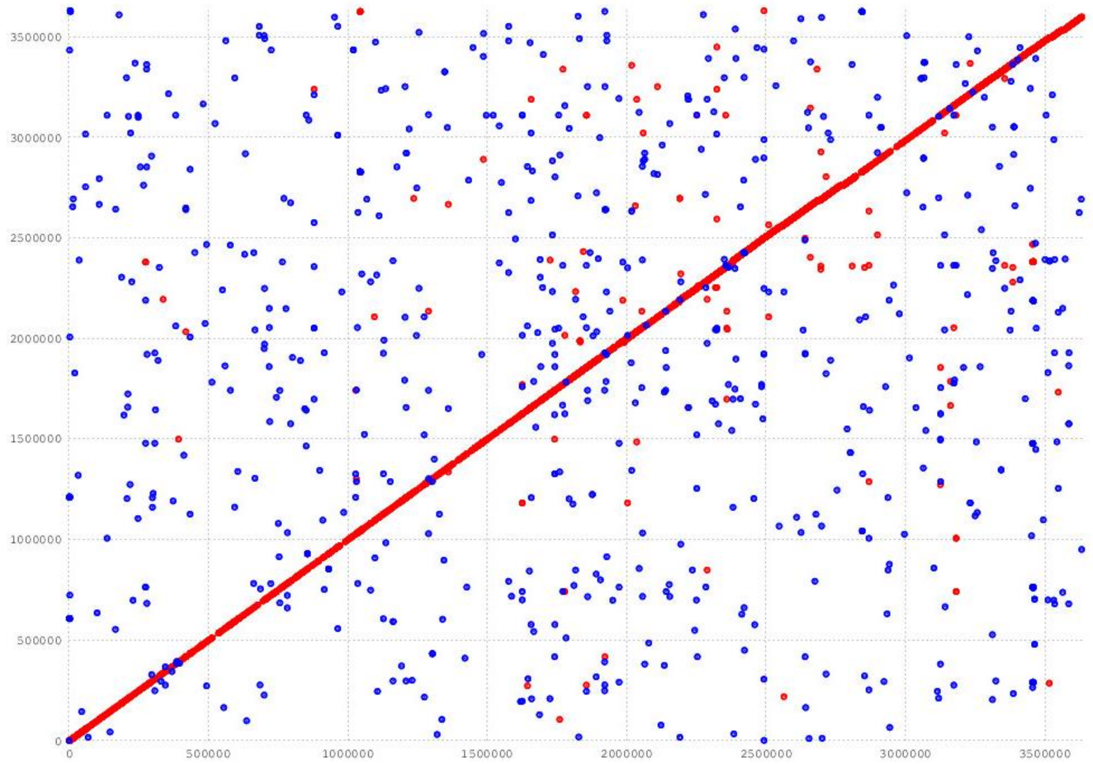

YALI1

YALI0

YALI0E – YALI1E Mummer Dot Matrix Comparison

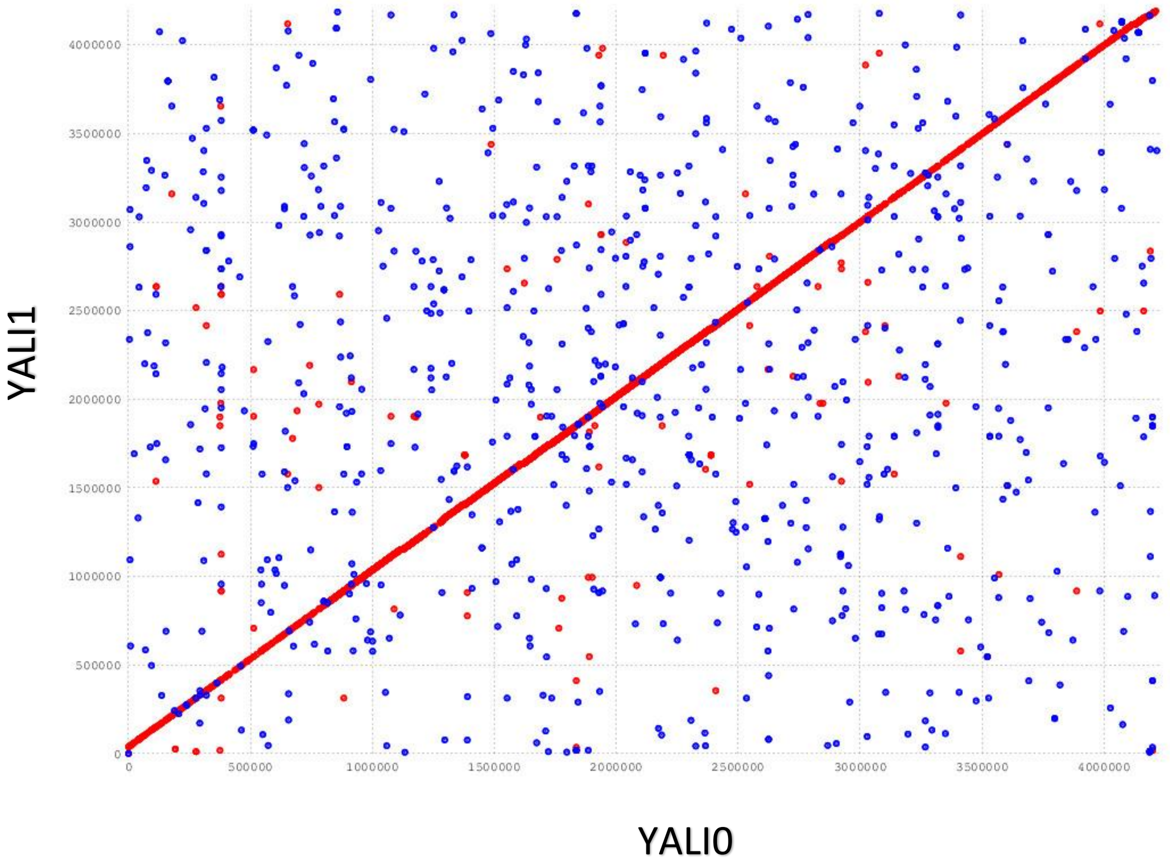

YALI0F – YALI1F Mummer Dot Matrix Comparison

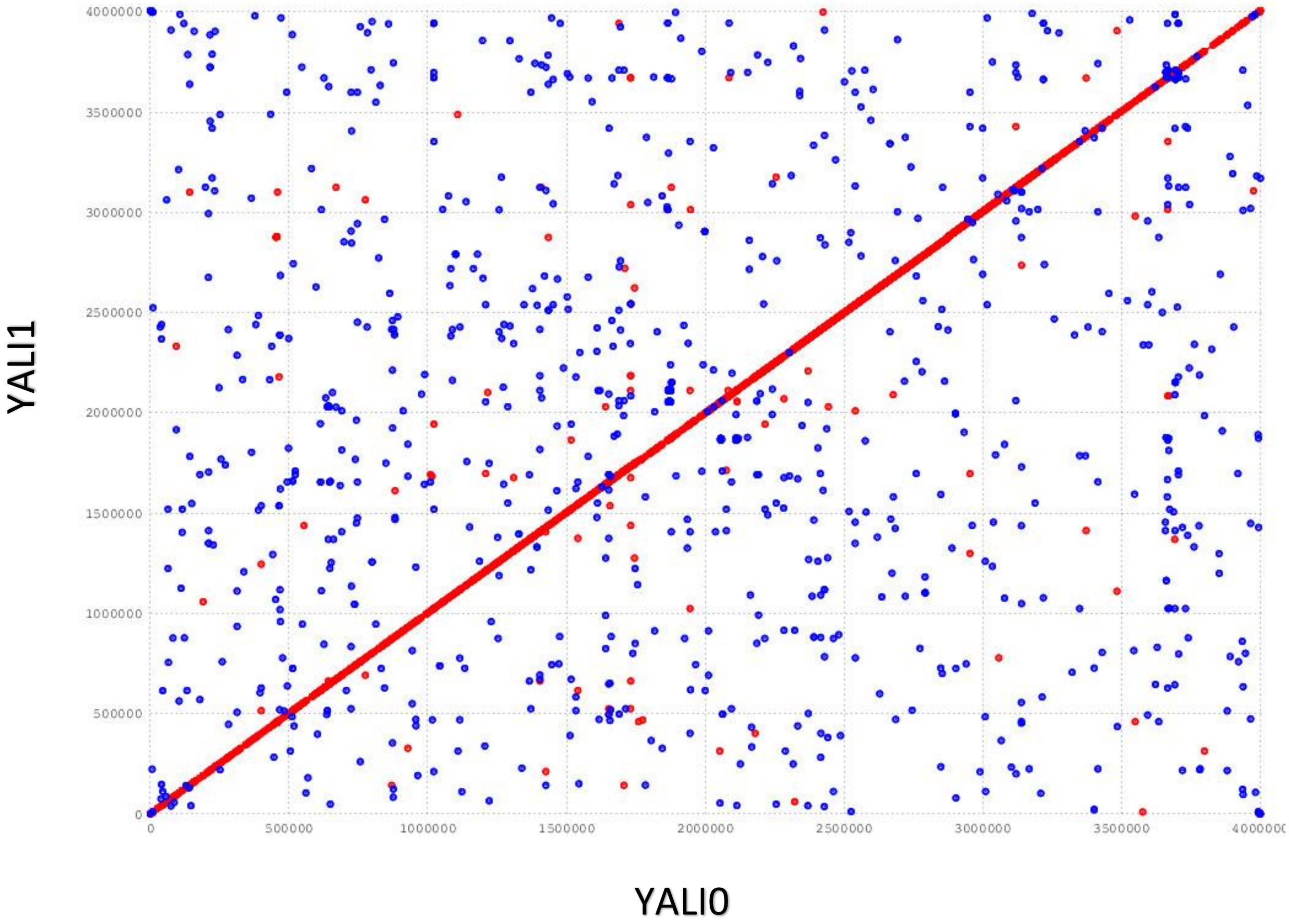

YALI0M – YALI1M Mummer Dot Matrix Comparison

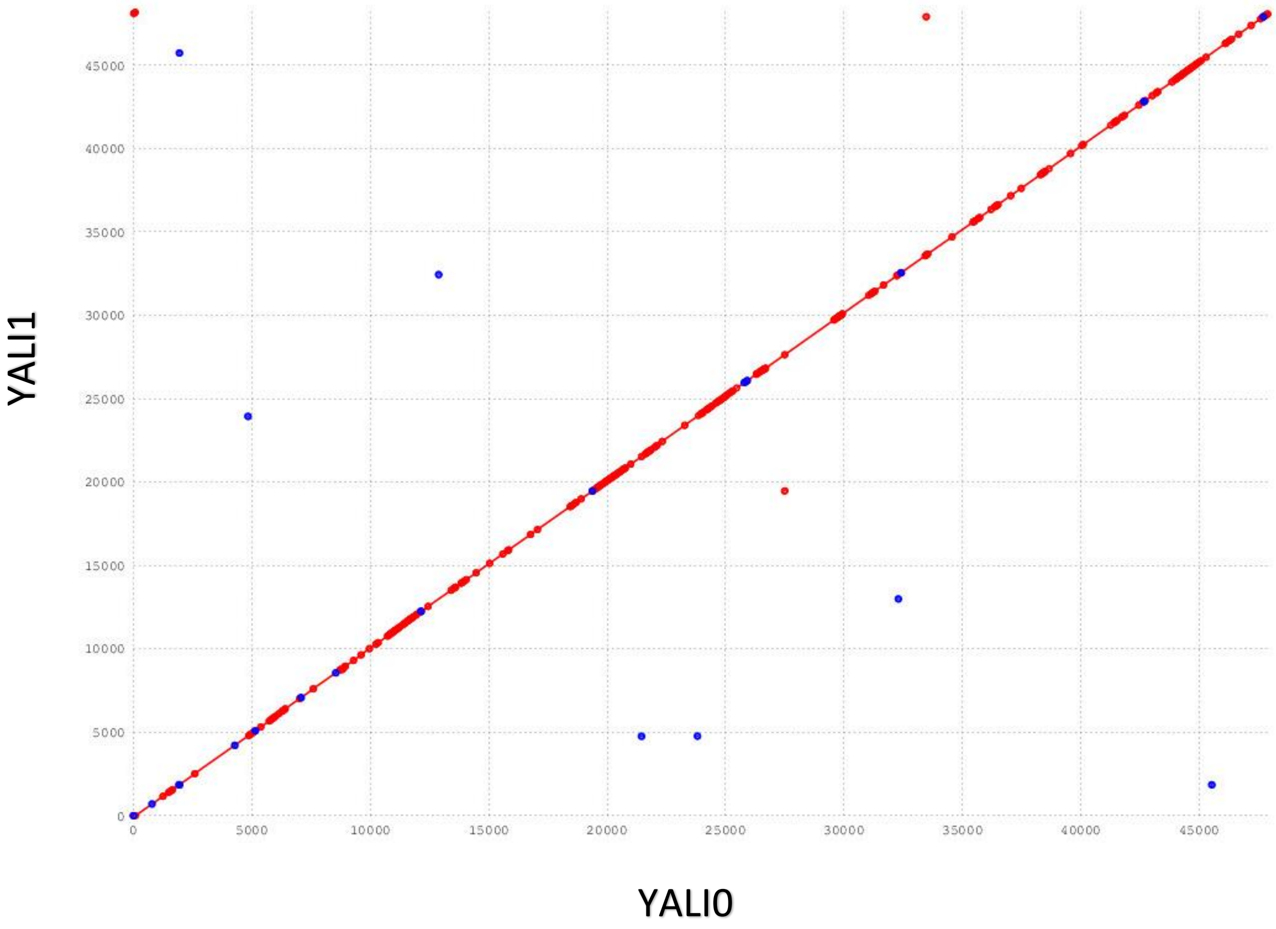

Supplement: S1 Fig — Genomes were globally compared to themselves and to each other by alignment of assemblies using a dot matrix program, MUMmer [79]. Red shows agreement in forward sense and blue in reverse sense. Chromosomes are compared in alphabetical order A to F and mitochondrial genome, M. Chromosome B, bottom panel shows expanded view of 51-kb inversion. Chromosome C, bottom panel shows expanded view of 54-kb repeat region. (PDF) [file pone.0162363.s001.pdf]
