# Supplementary figures and images for: Sequence Assembly of Yarrowia lipolytica Strain W29/CLIB89 Shows Transposable Element Diversity
Source: PLoS One. 2016 Sep 7;11(9):e0162363. doi: 10.1371/journal.pone.0162363 (PMC5014426; doi:10.1371/journal.pone.0162363)

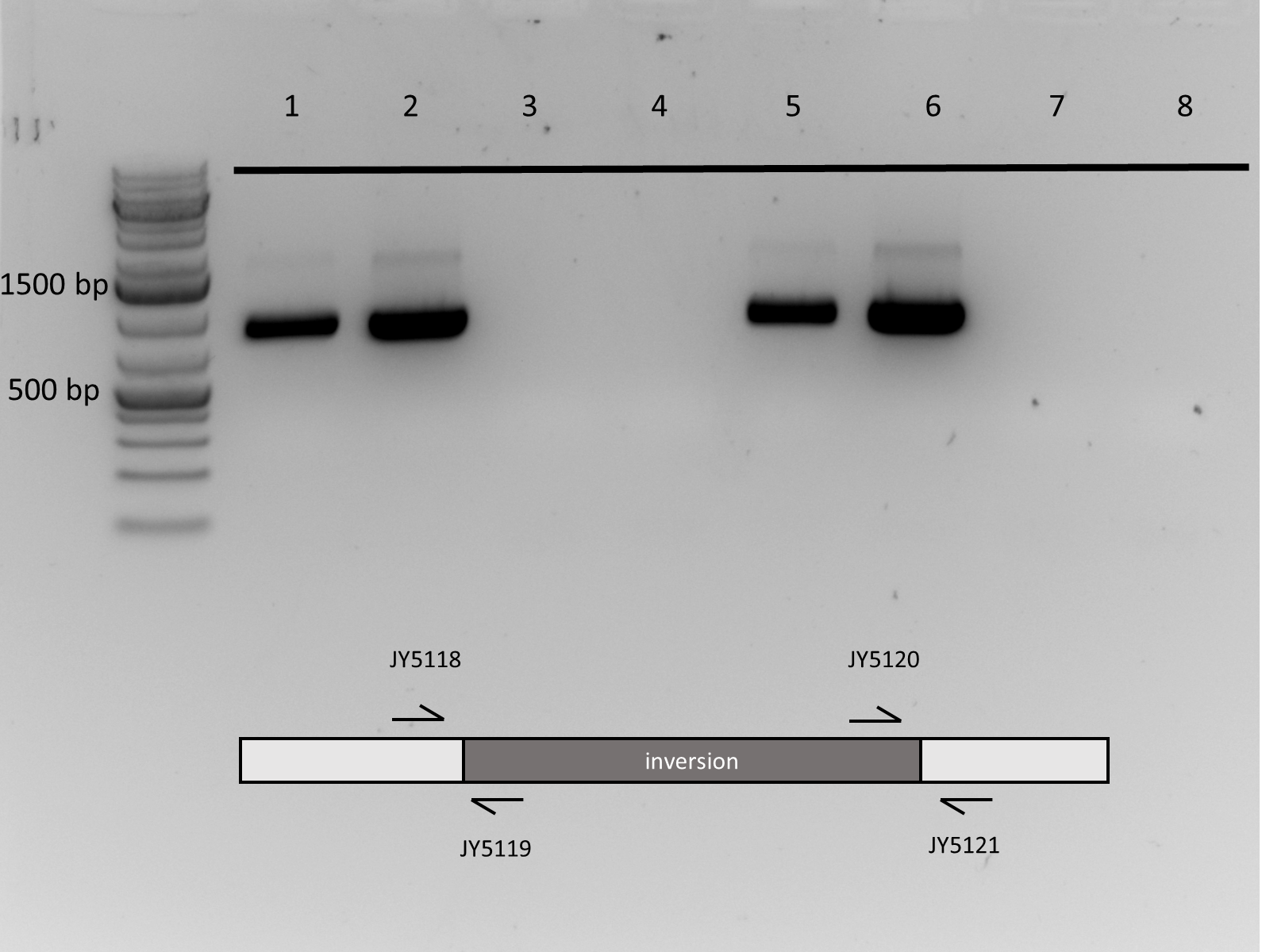

Supplement: S2 Fig — Primer pairs JY5118/JY5119 and JY5120/5121 were predicted to generate a PCR product in the CLIB89 YALI1 assembly. Lanes 1–4 utilized CLIB89 genomic DNA as template. Lanes 5–8 used PO1f genomic DNA as template. The primers used in the PCR reactions were detailed as follows: lane 1 and 5 (JY5118/JY5119), lanes 2 and 6 (JY5120/JY5121), lanes 3 and 7 (JY5118/JY5121), lanes 4 and 8 (JY5119/JY5120). (TIF) [file pone.0162363.s002.tif]
