# Supplementary material for: Sequence Assembly of Yarrowia lipolytica Strain W29/CLIB89 Shows Transposable Element Diversity
Source: PLoS One. 2016 Sep 7;11(9):e0162363. doi: 10.1371/journal.pone.0162363 (PMC5014426; doi:10.1371/journal.pone.0162363)
Supplement: S5 Table — (DOCX) [file pone.0162363.s007.docx]

Genes unique to CLIB89

| **Genes unique due to *in vivo* strain differences** | | | | |
| --- | --- | --- | --- | --- |
| **Hypothesized Protein Function** | **Chromosome** | **Start** | **Stop** | **Length (bp)** |
| Isopropyl Malate Dehydrogenase (*LEU2*) | YALI1C | 46,399 | 47,743 | 1,345 |
| Mating Type A Protein | YALI1C | 988,507 | 989,007 | 501 |
| Tyl3 Gag | YALI1C | 1,892,988 | 1,894,745 | 1,758 |
| Tyl3 Pol | YALI1C | 1,894,710 | 1,898,716 | 4,007 |
| OMP Decarboxylase (*URA3*) | YALI1E | 3,168,501 | 3,169,615 | 1,115 |

| **Genes unique due to assembly** | | | | |
| --- | --- | --- | --- | --- |
| **Hypothesized Protein Function** | **Chromosome** | **Start** | **Stop** | **Length (bp)** |
| Heat shock protein | YALI1A | 2,251,939 | 2,253,870 | 2,044 |
| Putative zinc finger protein | YALI1D | 3,602,943 | 3,604,748 | 1,806 |
| Putative lipase (serine esterase) | YALI1D | 3,605,391 | 3,606,710 | 1,320 |
| Argininosuccinate synthase | YALI1D | 3,606,992 | 3,608,481 | 1,490 |
| Putative CofD related protein | YALI1D | 3,609,608 | 3,612,159 | 2,552 |
| Mannosyltransferase | YALI1D | 3,614,723 | 3,616,132 | 1,410 |
| Probable glutathione S transferase protein | YALI1D | 3,617,980 | 3,620,213 | 2,234 |
| Heat shock protein | YALI1D | 3,621,902 | 3,625,550 | 3,649 |
| Heat shock protein | YALI1E | 11,566 | 13,641 | 2,076 |
| Putative cinnamyl alcohol dehydrogenase | YALI1E | 14,889 | 17,410 | 2,522 |
| Putative exonuclease | YALI1E | 31,359 | 29,843 | 1,516 |
| Ribsomal 50S protein | YALI1E | 31,812 | 32,720 | 909 |
