# Supplementary material for: Sequence Assembly of Yarrowia lipolytica Strain W29/CLIB89 Shows Transposable Element Diversity
Source: PLoS One. 2016 Sep 7;11(9):e0162363. doi: 10.1371/journal.pone.0162363 (PMC5014426; doi:10.1371/journal.pone.0162363)
Supplement: S1 Text — (DOCX) [file pone.0162363.s011.docx]

**S1 Text. Supplemental Materials and Methods**

**1. Sequencing datasets**

A total of four DNA and three RNA sequencing datasets were used in this study to assemble chromosome sequences for *Yarrowia lipolytica* CLIB89 or extract gene annotations from the assembled genome (Table 1A). This section provides a short description of their content.

**1.1. DNA sequencing datasets used to assemble the genome**

The genome de novo assembly is performed using four distinct DNA sequencing datasets described in this section. The first two datasets, YL97B and YL110 were generated using Illumina sequencing technology to generate short sequencing reads. The latter two datasets, YLP13 and YLP14, were generated using PacBio sequencing technology to generate short and long sequencing reads. The methods used to prepare the corresponding samples are provided in Supplementary Section 1.1. Specific information to each dataset is provided below.

- Dataset YL97B was generated on an Illumima HiSeq 2000 platform from a single-end 97 cycles run. The sequencing data were processed and demultiplexed using CASAVA 1.8.2. The dataset contains 14,951,623 single reads of length 97.
- Dataset YL110 was generated on an Illumina HiSeq 2500 platform from a paired-end rapid run (110 cycles per end). The data were processed using CASAVA 1.8.2. The dataset contains 389,608,406 paired reads of length 110 each. Quality scores for the Illumina runs are available upon request.
- Dataset YLP13 was generated on a PacBio RS platform using eight SMRT cells. The SMRT Analysis software provided by Pacific Biosciences was used to filter the initial sequencing data and extract the best sub-reads from each cell. The resulting dataset contained 157,966 reads of length varying between 8 and 22,569 bp. The mean read length for this dataset is 3,362 bp.
- Dataset YLP14 was also generated on a PacBio RS platform, using four SMRT cells. DNA was fractionated to enrich for longer fragment size. The best sub-reads were then extracted from each cell using the SMRT Analysis software provided by Pacific Biosciences. The resulting dataset contains 253,645 reads of length varying between 50 bp and 32,683 bp. The mean read length for this dataset was 5,642 bp. Quality scores for the PacBio analysis are available upon request.

**1.2. RNA sequencing datasets used to annotate the genome**

The genome annotation was generated as described below utilizing software to analyze transcript data for an independent purpose. These datasets are the result of three distinct experimental conditions, noted YL, CL, and NL respectively.

- The sample YL data were generated on an Illumina HiSeq 2000 platform from a single-end 97 cycles run. Combined together, the datasets contain 246,132,666 single reads of length 97.
- The CL and NL data were generated on an Illumina HiSeq 2500 platform from a PE 75 cycles. The sequences were demultiplexed using CASAVA 1.8.2. The CL data contained 118,181,936 PE of length 75 each. The NL dataset contains 118,638,382 paired-reads of length 75 each.

**2. *De novo* genome assembly**

The *Yl* genome was assembled using a *de novo* approach. The methods implemented during the various steps of the assembly combine in-house tools developed by the Institute for Genomics and Bioinformatics (IGB) of the University of California, Irvine (UCI) and third-party software available online. High-quality short reads in YL97B and YL110 datasets and lower-quality long reads in the YLP13 and YLP14 datasets were used alternately during to assemble each chromosome generally following a classical, hybrid genome assembly strategy The workflow can be summarized as four major steps: (a) *de novo* assembly of short, high-quality contigs using the reads in YL97B and YL110 using Velvet [[1](#_ENREF_1)]; (b) the detection of the overlapping junctions between short contigs using long reads in YLP13 and YLP14; (c) sequence refinement of junctions using Illumina and PacBio datasets; (4) assembly completion of unresolved gaps in the assembly and chromosome ends. Each section below provides a description of methods used during each.

**2.1. High-quality contigs assembly using Illumina short reads**

The first stage of the assembly consisted of de novo assembly of short contigs with the following properties: each contig had a unique sequence not repeated in another contig, did not contain repeated and unique fragments within the same contig, had a sequence confirmed with high-coverage by both sets of short reads (YL97B and YL110), and finally did not contain any position that could be a breakpoint. This combination of properties reduced and prevented assembly errors during later stages.

We used Velvet [[1](#_ENREF_1)] third-party software to obtain de Bruijn graphs-based assembly of the short reads in YL97B using a k-mer length of 31. A first filter was applied to the 4,400 resulting contigs to split any contig containing an uncalled base ‘N’ and remove any contig of length shorter than 200 bp. The single reads in YL97B and the paired reads in YL110 were aligned to the remaining contigs using Eland v2e (Illumina) and the alignment results were filtered to keep only reads fully mapped to a single contig with no mismatch allowed. Note that the short-read aligner used during this step did not affect the set of selected reads due to the filters applied. 54.69% of the reads in YL97B and 54.93% of the reads in YL110 were selected following this protocol. For each contig, the read coverage is computed for each position based on the selected mapping results. In addition, we computed for each position how many reads were overlapping but not ending at the position by applying the following rule: a read is overlapping a position if at least 5 bp of the read are located on each side of the corresponding position. For the paired-reads in YL110, the positions not sequenced between the two ends of the inserts are considered overlapped by the read and the positions overlapped twice due to short inserts are only counted as overlapped once by the read. Each contig was filtered by iteratively applying the following rules: (a) if two non-overlapping fragments of length at least 200 bp can be found such that one has a mean coverage value higher than the contig mean coverage value and the other one has a mean coverage value lower than the contig mean coverage value, the contig is split at the median position between the two fragments (b) if any position is such that the number of reads overlapping the position is lower than 90% of the number of reads mapped to the same position (excluding the contig ends), the contig is divided into two contigs at the corresponding position (c) end positions with a number of overlapping reads lower than 6 in the dataset YL97B or lower than 150 in the dataset YL110 are discarded (d) contigs shorter than 200 bp or with a mean coverage value lower than 20 for the reads in YL97B or 1,000 for the reads in YL110 are discarded.

The protocol described above resulted in 5,707 de novo contigs totalling 19,509,122 positions (94.94% of the final assembly length) and of average length 3,418 bp. Each contig position is covered in average by 39.74 reads in YL97B and 2,321 reads in YL110. The mean PHRED quality score of the sequencing reads mapped to these contigs with no mismatch is 35.09 for YL97B and 36.24 for YL110. Note that the protocol used provides with high confidence contigs with the properties listed at the top of this section. In fact, repeated sequences among the initial set of contigs are either short and do not need to be separated from the non-repeated sequences, as their instances are distinguishable using short-reads only, or are long, and in this case are directly discarded by the filtering step performed on the mapping results (no read mapped at different locations with no mismatch selected). Also, the initial contigs containing both an unique part of the genome and a repeated part of the genome are divided into two contigs either by the filtering step noted (a) above or by the filtering step (b) as in these cases the positions located on the repeated region at the limit with the unique region will have a ratio number of reads overlapping the position / number of reads mapped to the position significantly lower than 0.9. For the same reason, possible breakpoints are removed from the initial set of contigs.

**2.2. Contig scaffolding using PacBio long reads**

The contig scaffolding was performed using long reads obtained from PacBio sequencing (datasets YLP13 and YLP14) and using both the properties of the contigs described in the previous section and the properties of the reads themselves, notably the very high sequencing error rate that comes with the PacBio sub-reads (up to 20%). However, the mean length of these long reads was either similar (YLP13) or greater (YLP14) than the mean contig length (3,418 bp), indicating that PacBio reads on average are likely to spanmore than a single contig and therefore to provide useful scaffolding information.

The first stage of scaffold building utilizing PacBio long reads consisted ofextracting all possible matches between the PacBio long reads and the *de novo* contigs using BLAST [[2](#_ENREF_2)] third-party software and an e-value cut-off of 0.001. A total of 732,853 hits are found using this threshold. Each hit returned by BLAST was extended using a combination of in-house software and ClustalW [[3](#_ENREF_3)] third-party tool to include in the alignment all the bases in the corresponding read and contig that are expected to match together assuming the hit returned by BLAST is correct, i.e. extended on both sides until the end of the contig or of the read was reached, whichever occurred first. The percentage of identity between the mapped regions of the contig and the PacBio read was computed based on the ClustalW alignment result.

The second stage of our approach consisted of applying a first set of filters on the hits extracted during the previous stage in order to (a) remove the obviously irrelevant or unreliable hits based on the extended alignment results; (b) remove the hits that do not provide any scaffolding information; and (c) filter the expected ambiguous or conflicting cases. Filters in the category (a) removed all the hits where the percentage of identity computed from the extended alignment is lower than 75% and removed all the hits where less than 120 bp of the read or of the contig were included in the alignment. The filter denoted (b) above consists in removing all the hits involving a PacBio read not matched to any other contig. The filters in the category (c) are implemented for more complex cases. The 5,707 assembled de novo contigs (Section 2.1) have unique sequences but a simple BLAST alignment of these contigs against themselves revealed that 701 contig fragments had a very high sequence similarity with another fragment in the same set. These sequences can be expected to be matched to the same PacBio reads and at the same location and therefore needed specific filters. We applied the following rule for these conflicting hits: if after extending the BLAST alignments between a PacBio read and two conflicting contigs the percentage of identity between the read and one of the contigs becomes higher than the percentage of identity between the read and the second contig by at least 8%, the hit with the first contig was considered valid and the hit with the second contig was discarded, otherwise both hits were discarded. The same rule was applied in cases where a single read was matched twice by BLAST to the same contig with inconsistent positions or directions.

The third stage of our approach consisted of extracting for each *de novo* contig the list of all possible left (upstream) and right (downstream) mates based on the mapping results left after the first pass of filtering described above. For each candidate mate, the list of reads suggesting a junction between the two contigs is extracted together with the positioning information, including the distance between the two contigs on the read and their relative direction. Note that thanks to a relatively high number of very long reads, the average number of left and right mates extracted for each contig using this approach is 4.54, i.e. includes not only the closest mate of a contig, but also the next one in many cases. We applied at this stage a second set of filters to each group of reads suggesting the same junction. For each candidate junction, we removed all the reads previously selected with a corresponding positioning information inconsistent with the majority of the reads in the same group. More precisely, the relation position (upstream or downstream), the relative direction, and the distance between the two contigs were analyzed for each candidate junction and reads not matching the majority of reads selected for the same junction were discarded.

The last stage of our approach was the scaffolding of *de novo* contigs based on lists of left and right candidate mates previously extracted. Note that conflicting mates are expected at this stage for three different reasons. First, several *de novo* contigs were repeated in the genome, meaning that the left and right mates of each instance of a given repeat were grouped together in the same lists of candidate mates. Second, a few *de novo* contigs were overlapping, meaning that their expected distance computed from the PacBio reads was negative. Finally, long distance junctions (>/=4 kbp) led to some conflicts between the candidate mates due to the low number of reads available to confirm the junction and the error frequency of PacBio reads. In order to resolve these conflicts, we implemented the following scaffolding protocol. Repeated contigs were detected by checking for each contig, if two left candidate mates or two right candidate mates with at least eight PacBio reads confirming each junction were conflicting without being cases of overlapping contigs. Overlapping contigs were detected by simply checking that terminal sequences were identical. The threshold of eight reads guaranteed that only close mates were considered. Additionally, contigs with short read coverage greater than 60x for YL97B or 3400x for YL110 were considered as possibly repeated in the next steps. Contiguous repeated contigs of the form L,C^0^,...,C^n^,R where L and R were repeated contigs with conflicting left and right mates respectively and no conflict observed for their first right mate (C^0^ or R) and first left mate (C^n^ or L) respectively and where C^0^,...,C^n^ are contigs previously flagged as possible repeats due to their high coverage were scaffolded together and processed as a single contig in the next scaffolding steps.

The final scaffolds were obtained by repeating the steps described below starting for each scaffold from a non-repeated contig randomly selected among the set of contigs not already scaffolded. The closest right mate of the right-most contig in the scaffold (noted RMC afterward) validated by at least eight PacBio reads was selected as the best candidate to extend the scaffold. If there was no such mate, the scaffolding procedure was stopped. Otherwise, the repeat detection protocol described above guaranteed that no other candidate right mate with high coverage conflicted with the selected right mate. The selected contig was thus added to the scaffold and was therefore the RMC during the next iteration assuming this contig was not a repeat (see below). The remaining candidate right mates of the current RMC conflicting with the selected contig was then discarded from the list of candidate right mates for the RMC. After assembly completion, we observed that 100% of these conflicting mates were only suggested by one or two PacBio reads where the corresponding extended alignments passed the filters previously described by only a very short margin. In many cases, the RMC list of candidate right mates remaining after scaffolding the closest one could also be used to extend the scaffold further, but with a lower coverage and thus confidence. These lists were therefore only used afterward in two cases: (a) to check the consistency between the predicted RMC distant right mates and the next contigs added to the scaffold and (b) to assemble the various instances of the repeated contigs as explained below. Assuming a contig was added to the scaffold during the step described above, the next steps were as follows. If the contig added to the scaffold was not a repeated contig, the processing of the current RMC was considered completed and the added contig became the new RMC to process following the same protocol. Otherwise, the list of remaining candidate right mates of the current RMC was used to find the conflicting right mates of the repeated contig which was the correct one for this instance of the repeat. More precisely, we checked among all possible right mates of repeated contigs whether any of them was present in the list of distant right mates of the current RMC. With no exception, only one candidate was found for each instance of the repeated contigs. The junction between the two unique contigs separated by the repeated one was thus validated and the scaffold was extended to include both the repeated contig and its first right mate, finalizing the processing of the current RMC and setting the next RMC to be the last non-repeated contig added to the scaffold. The process is repeated while contigs can be added to the right side of the scaffold following this protocol. The same protocol is used to extend the left side of the scaffold. Two exceptions to this protocol were made during the assembly, both for cases where the closest mate found was too distant from the RMC to have eight reads validating the junction (6 kbp and 8 kbp respectively). The junctions were manually validated and later confirmed by the results obtained during the next assembly step (Section 2.3).

The scaffolding protocol described in this section resulted in eight scaffolds: five matching with the reference chromosomes noted *Yali0A*, *Yali0B*, *Yali0D*, *Yali0E*, and *Yali0F* in the reference assembly CLIB-122 [[4](#_ENREF_4)], one matching with the reference assembly of the mitochondrial genome noted *Yali0M* [[5](#_ENREF_5)] and two matching with the chromosome *Yali0C* of the CLIB-122 assembly [[4](#_ENREF_4)]. The last two scaffolds matched with chromosome C in the reference genome but were separated by 6.5 kbp. This sequence region was amplified by PCR and confirmed. Note that all of the 5,707 *de novo* contigs initially assembled were scaffolded during this stage of the assembly.

**2.3. Junction sequences assembly using long and short reads**

The junction sequences between the contigs scaffolded during the previous stage of the assembly are obtained following the protocol described in this section. This protocol involves both the PacBio reads confirming each junction obtained as described in the previous section and the short sequencing reads in the datasets YL97B and YL110.

For each junction, the set of PacBio reads covering both the junction and part of the adjacent contigs was used to extract an initial consensus sequence for the corresponding junction, and further optimized in a second stage using Illumina short reads. The long reads were trimmed at the junction level based on the extended mapping results described in Section 2.2. An additional 200 bp long fragment of each read was added to each side of the trimmed junction sequences in order to include part of the adjacent contigs in the consensus sequence. We use ClustalW [[3](#_ENREF_3)] to generate a multiple sequence alignment (MSA) of the resulting sequences and in-house software to extract a consensus sequence from the MSA. Positions in the MSA are selected for the consensus sequence every time enough reads confirmed the existence of the corresponding position and only when the agreement between the corresponding reads is high enough. These two parameters were not set to fixed values during the process in order to optimize them for each junction using the 200 bp on each side of the junction for which the target consensus sequence was known. The parameters were optimized using a greedy approach consisting in extracting a consensus sequence for a relatively large set of parameters, then computed for each resulting sequence its percentage of identity with the known contig sequences, and finally selected the consensus sequence with the highest percentage of identity with the adjacent contig sequences. Based on the sequence accuracy estimates computed during the process to select the best consensus sequence for each junction, the mean error rate of the consensus sequences was estimated to be around 8%, significantly lower than the error rate of a single PacBio read.

The junction sequences were further optimized using the short Illumina reads in the datasets YL97B and YL110. The single reads in YL97B were aligned to the scaffold sequences using Eland v2e (Illumina). Reads uniquely aligned with less than two mismatches with the scaffold sequences were discarded in order to obtain the subset of reads either not located on the scaffold sequences or mapped with several mismatches. A *de novo* assembly of the selected reads was performed using Velvet [[1](#_ENREF_1)] third-party software and the resulting contigs were compared to the junction sequences extracted from the PacBio reads. Every contig uniquely matched to a single junction with a percentage of identity higher than 90% is then used to correct the corresponding part of the junction sequence. Finally, the remaining junctions not optimized using this strategy are optimized by repeatedly aligning the paired reads in **YL110** to the scaffold sequences, selecting the paired-reads mapped such as at least one read is fully mapped to an optimized region of the scaffold with no mismatch and at least a part of the second read is mapped to a non-optimized part of a junction, and correcting the junction sequences based on a consensus assembly of the selected reads. Seven iterations of this process completed the error-correction of the junction sequences. The scaffold sequences were considered completed at this stage of the assembly and were not further modified.

**2.4. Assembly completion**

The last stage of the genome assembly involved evaluation of the possible gap on chromosome C mentioned in Section 2.2 and the possible missing positions at the chromosome ends. In fact, the eight assembled scaffolds all ended in one of the 5,707 *de novo* contigs initially assembled, meaning that any position located not included in these short contigs during was not represented in our assembly at this stage.

The two scaffolds matched with chromosome *Yali0C* of the CLIB-122 reference assembly [[4](#_ENREF_4)][4] were separated by a 6.5 kbp junction in this assembly not found during the contig scaffolding stage of our assembly. In order to determine if the two scaffolds are in fact parts of the same chromosome, the junction was checked by PCR experiment starting from unique sequences extracted from each scaffold end. The experimental results confirmed both the junction and the distance between the two scaffolds. The junction sequence was assembled following these steps. First, the paired-reads in **YL110** were repeatedly aligned to the scaffold ends to extend at each iteration the scaffold sequences based on a consensus assembly of the reads mapped to the scaffold ends with no mismatch and with at least one base located outside the scaffold limits. At each iteration, the PacBio reads not used during the previous stages of the assembly were aligned to each scaffold end. The iterations are stopped when three PacBio reads were successfully matched to both scaffold ends with alignment properties passing the filters described in Section 2.2. The missing part of the junction was obtained following the protocol described in Section 2.3. After this step, the six chromosomes of the Yarrowia genome and the mitochondrial genome were assembled with no gap.

Finally, the last stage of our assembly protocol consisted of extending the chromosome ends using the paired-reads in YL110 following the same iterative procedure as the one described in the previous paragraph for the missing junction on chromosome C. Iterations were halted when no additional sequence could be added either because the actual end seemed to have been reached or because two of the chromosomes terminated in similar sequences and therefore it was not possible to determine confidently whether a read belonged to one end or the other. After completion, the 12 chromosome ends in the assembly were compared with the corresponding ones in the CLIB-122 assembly. Four were found almost identical to the reference ones, five of them are found longer in our assembly for a total of 90.5 kbp not found in the reference assembly, and five of them were found shorter for a total of 36 kbp not found in our assembly. The latter five sequences not present in our assembly and the corresponding junctions with their respective chromosome ends were all found valid based on overlapping reads and PCR as described in Materials and Methods. In order to provide the most complete genome assembly possible, we included these sequences in the final genome assembly. The final length of each chromosome in the assembly is reported in Table 1B.

**References**

1. Zerbino DR, Birney E. Velvet: algorithms for de novo short read assembly using de Bruijn graphs. Genome Res. 2008;18(5):821-9. Epub 2008/03/20. doi: 10.1101/gr.074492.107. PubMed PMID: 18349386; PubMed Central PMCID: PMC2336801.

2. Altschul SF, Gish W, Miller W, Myers EW, Lipman DJ. Basic local alignment search tool. J Mol Biol. 1990;215(3):403-10. Epub 1990/10/05. doi: 10.1016/S0022-2836(05)80360-2. PubMed PMID: 2231712.

3. Larkin MA, Blackshields G, Brown NP, Chenna R, McGettigan PA, McWilliam H, et al. Clustal W and Clustal X version 2.0. Bioinformatics. 2007;23(21):2947-8. Epub 2007/09/12. doi: 10.1093/bioinformatics/btm404. PubMed PMID: 17846036.

4. Dujon B, Sherman D, Fischer G, Durrens P, Casaregola S, Lafontaine I, et al. Genome evolution in yeasts. Nature. 2004;430(6995):35-44. Epub 2004/07/02. doi: 10.1038/nature02579

nature02579 [pii]. PubMed PMID: 15229592.

5. Kerscher S, Durstewitz G, Casaregola S, Gaillardin C, Brandt U. The complete mitochondrial genome of yarrowia lipolytica. Comp Funct Genomics. 2001;2(2):80-90. Epub 2008/07/17. doi: 10.1002/cfg.72. PubMed PMID: 18628906; PubMed Central PMCID: PMC2447202.
