# Supplementary material for: Sequence Assembly of Yarrowia lipolytica Strain W29/CLIB89 Shows Transposable Element Diversity
Source: PLoS One. 2016 Sep 7;11(9):e0162363. doi: 10.1371/journal.pone.0162363 (PMC5014426; doi:10.1371/journal.pone.0162363)
Supplement: S2 Text — (DOCX) [file pone.0162363.s012.docx]

LOCUS yarrowia 8921 bp DNA linear 30-NOV-2015

FEATURES Location/Qualifiers

misc_feature 3154..3323

/note="5.8S rRNA"

misc_feature 3412..6322

/note="RDN25-1 "

misc_feature 1461..3082

/note="RDN18-1"

misc_feature 1254..1460

/note="5' ETS?"

misc_feature 6323..6521

/note="3' ETS?"

source 1..8921

/dnas_title="yarrowia rDNA sequence"

ORIGIN

1 GCTGTCTGTT TACCTCTAGA GTGGTTGGAG ACCATCAGAT GAGTTGTTTG AGATGAGTTG

61 TTTGTTGGGA CTAGTGTTCG AATCGACTAG TAAACGTCCG AGGTGAGTCT GACTGGTTGG

121 AGACCATCAG AGATGATCTT TATTGTGACT TAGTGTTTCA ACCGATGCAT TGTTTCTTTG

181 TGGCGGTCCC GGACTTGTCC GGAGGGTGTT CGAGAGTCAG CTGTAAGTCT GCGCTTGCTC

241 TGTGTGCTGT CTGTTTCTTC TGACCGGTTG GAGACCATCA GAGATGATCT TTATTGTGAC

301 TTTGTGTTTC ATCCTGATGC ATTGTTTCCT TGTGGCGGTC CCGGACTTGT CCCGGTGCAG

361 TCTGTTTCTC ACTGGTTGGA GACCATCAGC TACCTGTTTC ATCTTGATCC ACAGAATGCG

421 CAATATTCAT CTCACACGAC TGAAAACAAG TCCATTCGAT TTATCTCGGT CGATAACCCC

481 CAGTTGCCAT CATTCCAGTA CATTATTCAT TGATAGTAAC ATCCGCCACT TTCCAGTTAT

541 CGTATCTCAC ATCACCAAGT CGTGGAGGTT GGGAGGTGAG GTTGGAAGTT TAAACGAGGA

601 ACAAAGAAGA TCAACAAAGA CTTGTACGAG GCTAACCAAG TTTAACTTGA ATAAGGCCAG

661 GGACGGTTAG TTGGGTAATT CGGCTTGAAA TTGAGATAAT ACGGATAAAA GTCGAATTTA

721 ATATGATTAA ACGTATTCTG CGTCGATTTT GATGCTATAG CCTCATTTCG ATGTTGTTCT

781 GTGCACTGGT TTATTAAGTC CGGTTATGTC GAAGCATCCT ATATGAATGC CATATGATGT

841 ACACAAAGAG TTGGGAGTGA TATTAGAGTG TGTAAGGTGC ATGACTAAGC TTAAAAGGGT

901 ACAAAAGGGA GATGATTGAT GATCTAAGCG TGGTGAGAGA AGACAAAGTG TATGAGTTGA

961 GAGGGTGAAG ATGAAGAGGA TGAAGAGGAT AAAGAGGGAT TGAGCCGGCA TTGCTGTCGA

1021 CAAGGGGAAT GCCAGTTCCT GTGAGTTTGT GGGGACAACC TGTGTGAGTT GTGGTTTCCG

1081 GTGAAGTTGA CGTGGGAGAC GACCCGGTTG ACGACCTTGA CGACCAATGA CACCGATGAC

1141 ACAATGACCT GACAACGACC GGAGACCGTT GCTACACTGA GAACTCGAAA CACATATACC

1201 ACTACCTCAC CGCGATCCCA AAACCTCCCC CCCGCATCCA TCACATGGGG AAACCCAGAC

1261 ACAGCGGTGT TATTGCACTA CATTCTTCAG AATTAGTGGG TTGCTAGACA CTCTCTACAA

1321 CACATTGGCA ATGTACTGCC TTTCAATGGA AAAAAGTACA TGAGAGAACT CGTTGATCTC

1381 TCTACAAACA TCAACAATAC TTATCTGGTT GATCCTGCCA GTAGTCATAT GCTTGTCTCA

1441 AAGCTAAGCC ATGCATGTCT AAGTATAAAC AATTATACAG TGAAACTGCG AACGGCTCAT

1501 TAAATCAGTT ATCGTTTATT TGATAGTTTT CTACATGGAT AACCGTGATA ACTTCAGAAC

1561 TAATACATGA CAGCCTTCTG GCGTATATAT TAGATACAAA CCAACAGTAT GGTGATTCAT

1621 AATATCTTGT CGAACCGATC TTCGGTGTAT CATTCAAATT TCTGCCCTAT CAACTGTCGA

1681 TGGTAGGATC GTGGCCTACC ATGGTAACAA CGGGTAACGG GGAATCAGGG TTCTATTCCG

1741 GAGAGGGAGC CTGAGAAACG GCTACCACAT CCAAGGAAGG CAGCAGGCGC GCAAATTACC

1801 CAATCCTGAC ACAGGGAGGT AGTGACAATA TATAACGATC CGGGGCTCTT TGAGTTTCGG

1861 AATTGGAATG AGTACAATTT AAACACCTTA ACGAGGAACA ATTGGAGGGC AAGTCTGGTG

1921 CCAGCAGCCG CGGTAATTCC AGCTCCAATA GCGTATATTA ATGTTGTTGC AGTTAAAAAG

1981 CTCGTAGTTG AAATTGGGCG GGCTATTAGT TTAGGCCGCT TCAGGAAGAA CTTCTTCCAG

2041 TTACTTTGAA AAAATTAGAG TGTTCAACGC AGGTTTCGCC TGAATATATT AGCATGGAAT

2101 AACATAACAC GACGAGGGTC CATTTTGTTG GCTTGCAAAC CCACGTAATG ATTAATAGGG

2161 ACAGTCGGGG GCGTCAGTAT TGTGTTGTCA GAGGTGAAAT TCTTGGATTT ACACAAGACT

2221 AACTACTGCG AAGGCATTCG CCAAGGATGT ATTCATTAAT CAAGAACGAA AGTTAGGGGA

2281 TCAAAGATGA TCAGATACCG TCGTAGTCTT AACCGTAAAC TATGCCGACT GAGAATGGGT

2341 ACCGCTTATA CGGTATCCGC GCTCTACGAG AAATCAAAGT GATCAGGTTC TGGGGGGAGT

2401 ATGGTCGCAA GGCTGAAACT TAAAGGAATT GACGGAAGGG CACCACCAGG AGTGGAGCCT

2461 GCGGCTTAAT TTGACTCAAC ACGGGGAAAC TCACCAGGTC CAGACACAAT AAGGATTGAC

2521 AGATTGATAG CTCTTTCTTG ATTTTGTGGG TGGTGGTGCA TGGCCGTTCT TAGTTGGTGG

2581 AGTGATTTGT CTGCTTAATT GCGATAACGA ACGAGACCTT GACCTACTAA ATAGCTCTAC

2641 TTGCGATTGC AGGTAGCTAG CTTCTTAGAG GGACTATCTA TTACAAGTAG ATGGAAGTTC

2701 GAGGCAATAA CAGGTCTGTG ATGCCCTTAG ACGTTCTGGG CCGCACGCGC GCTACACTGA

2761 CGGAGCCAGC GAGTCGACCA AGCCCGAGAG GGCTAGGTAA TCTTGTGAAA CTCCGTCGTG

2821 CTGGGGATAG AGCATTGCAA TTATTGCTCT TCAACGAGGA ATTCCTAGTA AGCGCAAGTC

2881 ATCAGCTTGC GTTGATTACG TCCCTGCCCT TTGTACACAC CGCCCGTCGC TACTACCGAT

2941 TGAATGGTTT AGTGAGACCT TGGGAGGGCG AGATGAGGGG GGCAACCCCT TTTGAACATC

3001 CAAACTTGGT CAAACTTGAT TATTTAGAGG AAGTAAAAGT CGTAACAAGG TTTCCGTAGG

3061 TGAACCTGCG GAAGGATCAT TATTGATTTT ATCTATTTCT GTGGATTTCT ATTCTATTAC

3121 AGCGTCATTT TATCTCAATT ATAACTATCA ACAACGGATC TCTTGGCTCT CACATCGATG

3181 AAGAACGCAG CGAACCGCGA TATTTTTTGT GACTTGCAGA TGTGAATCAT CAATCTTTGA

3241 ACGCACATTG CGCGGTATGG CATTCCGTAC CGCACGGATG GAGGAGCGTG TTCCCTCTGG

3301 GATCGCATTG CTTTCTTGAA ATGGATTTTT TTAAACTCTC AATTATTACG TCATTTCACC

3361 TCCTTCATCC GAGATTACCC GCTGAACTTA AGCATATCAA TAAGCGGAGG AAAAGAAACC

3421 AACAGGGATT GCCTCAGTAA CGGCGAGTGA AGCGGCAAAA GCTCAAATTT GAAACCCTCG

3481 GGATTGTAAT TTGAAGATTT GGCATTGGAG AAAGCTAACC CAAGTTGCTT GGAATAGTAC

3541 GTCATAGAGG GTGACAACCC CGTCTGGCTA ACCGTTCTCC ATGTATTGCC TTATCAAAGA

3601 GTCGAGTTGT TTGGGAATGC AGCTCAAAGT GGGTGGTAAA CTCCATCTAA AGCTAAATAC

3661 TGGTGAGAGA CCGATAGCGA ACAAGTACTG TGAAGGAAAG GTGAAAAGAA CTTTGAAAAG

3721 AGAGTGAAAT AGTATGTGAA ATTGTTGATA GGGAAGGAAA TGAGTGGAGA GTGGCCGAGG

3781 TTTCAGCCGC CCCTCGTGGG CGGTGTACTG CCGACGCCGA GTCATCGATA GCGAGACGAG

3841 GGTTACAAAT GGGAGCGCCT TCGGGCGTTC TCCCCTAACC CTCCACACTG CCACCGACGA

3901 CATAATCCAC CCATTTCACC CGTCTTGAAA CACGGACCAA GGAGTCTAAT GGATATGTGA

3961 GTGTTAGGGT GGCAAACCCC AGCGCGCAAT GAAAGTGAAT GGATTCGTTC AGAATCGACC

4021 GAACTTGATT ATTATGACAG TTTTGAGTAA ACACATCCAT TGGGACCCGA AAGATGGTGA

4081 ACTATGCCTG GATAGGGTGA AGTCAGAGGA AACTCTGATG GAGGCTCGTA GCGGTTCTGA

4141 CGTGCAAATC GATCGTAGGA TCTGGGTATA GGGGCGAAAG ACTAATCGAA CCATCTAGTA

4201 GCTGGTTCCT GCCGAAGTTT CCCTCAGGAT AGCAGAAGCT CATATCAGTT TTATGAGGTA

4261 AAGCGAATGA TTAGAAGTAT TGGGGGCGAA ATGCCCTCGG CTTATTCTCA AACTTTAAAT

4321 ATGTAAGAAG CCTTGGTTAC TTAATCGAAC CGTGGCTACG AATGAAGAGC TTCTAGTGGG

4381 CCATTTTTGG TAAGCAGAAC TGGCGATGCG GGATGAACCG AACGTGGAGT TAAGGTGCCG

4441 GAATACACGC TCATCAGACA CCACAAAAGG TGTTAGTTTA TCTAGACAGC CGGACGGTGG

4501 CCATGGAAGT CGGAATCCGC TAAGGAGTGT GTCACAACTC ACCGGCCGAA TGAACTAGCC

4561 CTGAAAATGG ATGGCGCTTA AGCGTGTTAC CTATACTCTA CCGAGAGGAG GTTTCCTCTC

4621 GAGTAGGCAG GCGTGGGGGT TGTTGAGAAG CGTTGGCCGT GAGGCTGCGT CGAACGGCCC

4681 CTAGTGCAGA TCTTGGTGGT AGTAGCAAAT ATTCAAATGA GAACTTTGAA GACTGAAGTG

4741 GGGAAAGGTT CCGTGTGAAC AGCAGTTGGA CACGGGTAAG TCGATCCTAA GGGGTGGCAT

4801 AACTGTCGCG TACGGCCCGA TAAGGGCCTT CTCCAAAAGG GAAGCCGGTT GAAATTCCGG

4861 CACTTGGATG TGGATTCTCC ACGGCAACGT AACTGAATGT GGGGACGGTG GCACAAGTCT

4921 TGGAAGGAGT TATCTTTTCT TTTTAACGGA GTCAACACCC TGGAATTAGT TTGTCTAGAG

4981 ATAGGGTATC GTTCCGGAAG AGGGGGGCAG CTTTGTCCCC TCCGATGCAC TTGTGACGCC

5041 CCTTGAAAAC CCGCAGGAAG GAATAGTTTT CACGCCAAGT CGTACTGATA ACCGCAGCAG

5101 GTCTCCAAGG TGAACAGCCT CTAGTTGATA GAATAATGTA GATAAGGGAA GTCGGCAAAA

5161 TAGATCCGTA ACTTCGGGAT AAGGATTGGC TCTGGGGGTT GGTGGATGGA AGCGTGGGAG

5221 ACCCCAAGGG ACTGGCGGCT GGGCAACTGG CAGCCGGACC CGCGGCAGAC ACTGCGTCGC

5281 TCCGTCCACA TCATCAACCG CCCCAGAACT GGTACGGACA AGGGGAATCT GACTGTCTAA

5341 TTAAAACATA GCTTTGCGAT GGTTGTAAAA CAATGTTGAC GCAAAGTGAT TTCTGCCCAG

5401 TGCTCTGAAT GTCAAAGTGA AGAAATTCAA CCAAGCGCGG GTAAACGGCG GGAGTAACTA

5461 TGACTCTCTT AAGGTAGCCA AATGCCTCGT CATCTAATTA GTGACGCGCA TGAATGGATT

5521 AACGAGATTC CCACTGTCCC TATCTACTAT CTAGCGAAAC CACAGCCAAG GGAACGGGCT

5581 TGGCAGAATC AGCGGGGAAA GAAGACCCTG TTGAGCTTGA CTCTAGTTTG ACATTGTGAA

5641 GAGACATAGG GGGTGTAGAA TAAGTGGGAG CTTCGGCGCC GGTGAAATAC CACTACCCTT

5701 ATCGTTTCTT TACTTATTTA GTAAGTGGAA GTGGTTTAAC AACCATTTTC TAGCATTCCT

5761 TTCCAGGCTG AAGACATTGT CAGGTGGGGA GTTTGGCTGG GGCGGCACAT CTGTTAAAAG

5821 ATAACGCAGA TGTCCTAAGG GGGACTCAAT GAGAACAGAA ATCTCATGTA GAACAAAAGG

5881 GTAAAAGTCC CCTTGATTTT GATTTTCAGT GTGAATACAA ACCATGAAAG TGTGGCCTAT

5941 CGATCCTTTA GTTGTTCGGA GTTTGAACCT AGAGGTGCCA GAAAAGTTAC CACAGGGATA

6001 ACTGGCTTGT GGCAGTCAAG CGTTCATAGC GACATTGCTT TTTGATCCTT CGATGTCGGC

6061 TCTTCCTATC ATACCGAAGC AGAATTCGGT AAGCGTTGGA TTGTTCACCC ACTAATAGGG

6121 AACGTGAGCT GGGTTTAGAC CGTCGTGAGA CAGGTTAGTT TTACCCTACT GATGGACTTG

6181 TTGCAATAGT AATTGAACTT AGTACGAGAG GAACAGTTCA TTCGGATAAT TGGTGTTTGC

6241 TGCTGTCTGA TCAGGCAATG CAGCGAAGCT ACCATCCGCT GGATTATGGC TGAACGCCTC

6301 TAAGTCAGAA CCCATGCTAG AAGTGATGAA TTAGGGGGTA GGATGGATAT GAATAAGTAT

6361 CGCAGTACCG GAGGGGGAGT TTGGGTGGAT AAGGAAATTG TCTGCTCTTC CCTGATTGTA

6421 AAGATTTACC CTTGTGAAAT CCATTGTAGA CGACTTTAGT ATGCGACGAG GTATTGTAAG

6481 TAGTAGAGTA GCCTTGTTGT TACGATCTAT TGAGATTAAG CCTTTGTTGT TTAGATTCGA

6541 GGCGAGGCGC GTACTTTACG AGGCGCGTAG ACAGCGTAGC TGTTGAAGTG CCGAGCAACA

6601 TGATGAGGCA CGTAGACAGC GTAGCTGTTG TAGTGCCGAG CATACTTTTC GAGGCGCGTG

6661 CTTGACGAGG CACTTGACGA GGCACTTGAC GAGGCGCGTG CTTGACGAGG CACTTGACGA

6721 GGCACTTGAC GAGGCGCGTG CTTGACGAGG CACTTGACGA GGCGCGTGCT TGACGAGGCA

6781 CTTGACGAGG CGCGTGCTTG ACGAGGCACT TGACGAGGCG CGTGCTTGAC GAGGCACTTG

6841 ACGAGGCTCG TAGACAGCGT GACTGTTAAA GTGCCGAGTG GTGATGGATC AGCTGTCACT

6901 GACGCTCACA GTCAAGTCTG ACCACGTCAC GTAGGATTCC AAGTCCTGAC TAAGGACCAT

6961 GTGCTGACGT TTGCAGAACA TGGTATGTGT CGAAGACTGA TTATTTTGTT CTTGAGTGTT

7021 CAAGATCTTA AAGAGTTGAA AAGACGATAG TGCCTGTGAT TGAGGTTGTT GTGTTGTTTC

7081 AACGGAGTTG AATTTGCGGG GGGATGTTTT TCACGTAATG GAGGGCCTGA CGGGGTTCAT

7141 CCGGAGGTCT GACGTCTAGA GGTAAATGTT GTGGTGTCTG TCCTTGTAGA CAGGTTGGAG

7201 ACTGTCCGGA CGTCTAGAGG TAAATGTTCT GGTGTCTGTC CTTGTAGACA GGTTGGAGAC

7261 TGTCCGATAC ATCCTTTGTT GTGATTAGTG TTTCAGCTGT GAGTCTGCGC TGTTTGTGTC

7321 CTTGTAGACA GGTTGGAGAC CATCACATGT CTTTTGTTGG GACTTGTAAA CATTTGTTTG

7381 TGTTCACGTT TGAGGGGCCG TCTGTGTCCT TGTAGACAGG TTGGAGACCA AAGAGGTCCT

7441 TGTTGGGACT AGTAGACCTT GCCTTGTGTT CAAGAGTCGA TTAGTAAACG TCTGAGGTGC

7501 AGTCTGTGTC CGTCCAGACT GGTTGGAGAC CATCAGATTG TTGTTTGTTG TGACTTTGTG

7561 TTTCAAACCG GTGGATTGTG ACATTGTGAC GACGGTGCCG GACTTGTAGA GGCAGGTTTG

7621 AATGTTTCTG GTTGTCTGTG TCCTTGTAGA CAGGTGGAGA CCGTCAGATC GTTGTTGTGA

7681 CTAGTTGACA TTTGTTTGTG TTCACGTTTG AGGTGTGTCT GTGTCCTTCT TAACAGCTTG

7741 GATACCATCA GATTGTTGTT TGTTGTGACT TTGTGTTTCA AACCGGTGGA TTGTGTCATT

7801 GTGACGGTGC CGGACTTGTA GAGTCAGGTC TAAACGTCTC TGCTTGCCTC TGTGGTGCTT

7861 TCTGTGTCCT TCTAGACTGG TTGGAGACCA TCACATGTCT TTTGTTGTGA CTAGTCGACA

7921 TTTGTTTGTG TTCACGTTTG AAGTGCGTCT GTGTCCTTGT AGACTGGTTG GAGACCATCA

7981 GATGAGTTGT TTGTTGTGAC TTTGTTTCAA ACCGGTGGAT TGTTTCGTTG TGACGGTCCC

8041 GGACTTGTCC CGGAGGGTGT TCTAGAGTCA GCTGTAAGTC AGCGCTTGCT TCTGTGGTGC

8101 TGTCTGTTTC TTCTGACCAG TTGGAGACCA TCAGAGATGA TCTTTATTGT GACTTTGTGT

8161 TTCAAACTGG TGGATTGCTT CCTTGTGATG GACCGGACTT GTCCCGGAGG GTGTTCTAGA

8221 GTCAGCTGTG AGTCTGCGCT TGCTTCTGTG CTGTTTGTGA CCTTCTAGAC TGGTTGGAGA

8281 CCATCAGATA CATCCTTGTT GTGACTTAGT GTTTCTACCC GGTGGATTGT CTCCTTGTGA

8341 TGGACTGGAC TTGTTCCGAA GGGTGTTCGA GAGTCAGCTG TGAGTCTGCG CTTGCTTCTG

8401 TGGTGCTGTC TGTTTCTTCC GACTGGTTGG ATACCATCAG ATGAGTTGTT TGTTGGGACT

8461 TGTTGTGTAC ACGTTTGAAG TGTGTCTGTG TCCTTGTAGA GTCAGCTGTG AGTCTGCACT

8521 TGCTTCTGTG GTGTTTGTGT CCGTCCAGAC TGGTTGGAGA CCATCAGGTC GTTGTTTGTT

8581 GTGACTTTGT GTTTCAAACC GGTGGATTGT GTCATTGTGA TGGACCGGAC TTGTCCCGGA

8641 GGGTGTTCTA GAGTCAGCTG TGAGTCAGCG CTTGCTTCTG TGGTGCTGTC TGTTTCTTCC

8701 GACCGGTTGG AGACCATCAG AGATGATCTT TATTGTGACT TAGTGTTTCA TCCTGATGCA

8761 TTGTTTCCTT ATGGCGGCCC CGGACTTGTC TCGGTGGGTG TTCTAGAGTA AAAGTCTGAT

8821 GTGCAGTCTG TTTCCCACTG GTTGGAGACC ATCAGATCGT TGTTTGTTGG GACTAGACGT

8881 TGCCTTGTGT TCTAGTGGGG TTGGTAGTAT ACTTCTGACC T

//
