# Supplementary material for: Sequence Assembly of Yarrowia lipolytica Strain W29/CLIB89 Shows Transposable Element Diversity
Source: PLoS One. 2016 Sep 7;11(9):e0162363. doi: 10.1371/journal.pone.0162363 (PMC5014426; doi:10.1371/journal.pone.0162363)
Supplement: S3 Text — (DOCX) [file pone.0162363.s013.docx]

Ty3 Reverse Transcriptase: Identities = 203/490 (41), Positives = 288/490 (59), Gaps = 33/490

TIQSVEPNATDHSNKDTFCTLPVWLQQKYREIIRNDLPPRPADINNIPVKHDIEIKPGARLPRLQPYHVTEKNEQEINKIVQKLLDNKFIVPSKSPCSSPVVLVPKKDGTFRLCVDYRTLNKATISDPFPLPRIDNLLSRIGNAQIFTTLDLHSGYHQIPMEPKDRYKTAFVTPSGKYEYTVMPFGLVNAPSTFARYMADTFRDLRFVNVYLDDILIFSESPEEHWKHLDTVLERLKNENLIVKKKKCKFASEETEFLGYSIGIQKIAPLQHKCAAIRDFPTPKTVKQAQRFLGMINYYRRFIPNCSKIAQPIQLFICDKSQWTEKQDKAIDKLKDALCNSPVLVPFNNKANYRLTTDASKDGIGAVLEEVDNKNKLVGVVGYFSKSLESAQKNYPAGELELLGIIKALHHFRYMLHGKHFTLRTDHISLLSLQNKNEPARRVQRWLDDLATYDFTLEYLAGPKNVVADAISRAVY

Ty3 Integrase Core Domain: Identities = 55/120 (45), Positives = 74/120 (62), Gaps = 2/120 (2)

LDISMDFVTGLPPTSNNLNMILVVVDRFSKRAHFIATRKTLDATQLIDLLFRYIFSYHGFPRTITSDRDVRMTADKYQELTKRLGIKSTMSSANHPQTDGQSERTIQTLNRLLRAYAST

Ty3 Integrase C terminus, downstream of core domain: Identities = 73/218 (33), Positives = 117/218 (54), Gaps = 3/218 (1)

NIQNWHVYLPQIEFVYNSTPTRTLGKSPFEIDLGYLPNTPAIKSDDEVNARSFTAVELAKHLKALTIQTKEQLEHAQIEMETNNNQRRKPLLLNIGDHVLVHRDAYFKKGAYMKVQQIYVGPFRVVKKINDNAYELDLNSHKKKHRVINVQFLKKFVYRPDAYPKNKPISSTERIKRAHEVTALIGIDTTHKTYLCHMQDVDPTLSVEYSEAEFCQIPERTRRSILANFRQLYETQDNPEREEDVVSQNEICQYDNTSP

POL domain of new retroelement has homology to the previously reported yl16 retrotransposon in Y. lipolytica (YALI0E13288p2). Identities = 614/1172 (52%), Positives = 823/1172 (70%), Gaps 38/1172(3%)

Legend:
tRNA

LTR

GAG

Protease

RT

RNaseH

Integrase

Terminal direct repeats

TAGTTAGATATGACAACTAGTCGAAGCACAAACTGATGAAGATCCGCAAAAAGACCATATAACAGCAACTAGAACAGATATCTTCCCCGTCCCTTCAATTACTTGATCAGACAGTAGCAGATCTGGCTTTATCGTGCTACAGTACATACAGTACTTTCAGTCAATCTCCCTCTCCTCTTGTATATAACGGCCCATCGTCATCTATTGCAGTAGAACTACCACACGAAACCAACCACCACGTTTTCCAGGAACACGGTGGGACGTCGAGGACTTCACCAATAAAGAGATGTCATCAAGGGCTTCCGAATGAGCCCAAAAAATATGCAAATCTGGTTCTCGTCAAGGCAAAAAGGACAAAAAGGTCGTACCCGGAATCGAACCGGGGGTTACTGGAAGACGACTTATTAAGAGTCAGAATCCCAAGGTGATAACCGCTACACTATACAACCGTGAAGAAAAGGATGATTTTCTGGCAGGATCAACTGCCGACGTTCTGCGTGTTAAGGCAGATGCCATGACAAACTAGACAAAGAAACCGAACCCTTGATTTTTTGTAAGGACCAAAGTCGCTTACTAAGGGCTTGGGGGTGCCGGTCACGTGCAAGGGTGACTTGTTTCCCACCTGGGACGCACCCCGGGGGGGAGTATATAAGAAGGGACATTTATGTATGATTAAGAAACAAGTTATCACCAGCCTGGAACGTTATTACAGACAGCGAAGCAACGGTTAGATTAAGACAGCGACAAATTCTACCGACTGAGAACTTTCCGCAAGATAAGAGACGATCGGATCTTACATTTGGTAGCGATGCCCTCGGTTACGTTTCAGACAGAGAGACTTCAGTTTAAGACATCGATTCACCATACGAGGCACAAGATTGGGCACGTGGGGAATCCCAGATTCGATCGACGACACAGACTACGACGAGTCTTACGCAAGTTCTGATACGTTCGCAACAGCAGATTCAATGACAGTACCCCCCACAGGAACAGGCGTGGGCGGAGAGGGCGAGGTTGGCCATAACAACCAGGCGGGACAGGACGTCCCCCAGGACGAGCTCCAAGGTTTCAACGATGATTTTGAACTAAACGTTAACCTGGAGGAGGTGTCTGAGGCCGGGAGAGCGCGGATCGAGCGCACGACGGCTGTGATACAGGAGATGCAGGAGAGGCATGATCGGGAGATGGAAGCCGCTATACGAGCACTGCAGGACCGCGAGAAGGGCCTGAGCGCCAAGGAACCCCAGCGGGACGACAAAACGGAGCAGGATCGCTTCAATCGGAGCCGTCAGCTCGCCGATATGAAGAATTTCGGTAAGCACTTCTCGGGCAAGGAGAAGGACCGCGGCTTCGCCGTGCGCAACTTCCTGCAACGGCTGGACAACCGGTTCGAGGATCGGTATGTCCCGGTGGAACACCGAGCCGTGCTGGCCGCCGATGCCATGGAAGGTCCGGCCCAGGAGTGGTGGAGCACCTTGGCCAAGGAAACGCGGTCTGCCATGAAGGCCGATTGGGATCTGTACAAGAAACACGTGACCGCATATTTCACGCCTAAGGAGTATGCCTTGAACGCCGACACTCGGTTCTACAAGATGCAGGCTACTAGCAAGGAGACGACGGAGGCGTATGTGGCCAGGTTCCAGAGCGTGCTGCGTGGTATGGACTCCGAGCCGCCCATGACTATCATCGCTTCGATCTTCCTGCAGGGCATGAAGCCTGACTGGCAGAAGGGTGTGAAGATCGCCGCGGGTACCCGAGACTACCGAAAGATGACATTGGATCACCTGATCGAGTTGCTACAAGTACATTCTGGCAACCAGTTCGCTAGAGTGGGCGATTATATGGATGTCGACCTGGCTCAGACTTACGACACTGACTACGATGACTACGAGTCGGATCCAGAGGAGGCGCAGCTGATGAGGGCTCAAGCCAACGATTACGGAGGACGTCGGGGTCGTTACCGCTCGGGAGGACGGGGTGATGCCCGACCCCAGGGTCGTTCGGGAGGTTCCAACCGGGGCAACTCGGCCAACCGGAACAACCCGGGAGCCCAGACAGAGAAGAGCGGAGGCGATGAAGGGTGTTGGAACTGTCAGGAGTTAGGGCACACCCGACACCGCTGCCCTAAGCCCCGGACTGCTGCCTACGTCAGGCACCAGTTGGAGTATTGGACCCAACAGCTGGAGAAGGAGCAGGGAAAAGAGCAGAGGTAGTTGAGAACGAGACCTCAACTACCGTAGCATCCACAGATCTCGATCGTGAGGGTGCGGAAGCTCGATCTGAAATAGGTAACACAAAGTCAGTCGAGAAGACCGTACCCGTAGACTTGCCACAGCAAG*AGTCACCCGTGGAAGTGACACCGGAGGAACCCGAGCAGGAAACAGCGAAGAAGCGACGCAAGTCGACGGCTCAGAGGGCGGCGAAGAAGCGACGCAAGTCGACGGCTCAGAGGGCGGCGAAGAAGCGAGCGGCGAAAAGAGTCAAGAAAGCCGTCGCCAAGGCCACTAGGGAGTTGGAGTGGAAGGTGGAGGCCCGAAATGGGTGATGGAGGAGAAGAGTCGCCCGAGCCGACAGAGCTGCAGGTGCTCCGAACGCTCCGAGCGCCGAAGGTACAAGGGACCGTGCCGGAGGAATCAGGCGCCGATCTGGAAGGAGAGGAAGATGACGACGTGGAGGGCAATATCGAAGTGTTAGCCTTGTCATACGATGTCAATGGACAACGGCTGAAGGCCCTCCTGGACAGCGGTGCTAGCGCGTGCTTCAT*TGCGGCAAAGACGGCTGCACGATTGGGGCTCGAGACACAGGCGTGCACACCAAAGAAAGTGGTGTCGGTGCACGGCTCCGAGGTCTGCAAGCTGACCGCCCGGGTGCCGGTGAAAGCTGGCAAGTGGATGAAGCATGTGAAGGCGTACGTCCTCCAGCAGATGCACGGACAAGAGGTGTTGTTGGGTATGCCGTTCTTTATCAAGTATCATAGGTACATAGAGTGGGGCAGTAGAGAATTCCTACCTCCCGGCTGTGACCCCTCAGCAACCGAGAGAGGGGAGGAGGAGTTCCTGAATGCGTACACGGTGTCGTTGAACGACGCCAAGAGAGCCGTAGTCAGAGAGAGTGGAGAGATGTTTGTATGCTTCGTTAAGGAGCAGAGCGTAGACGCTACAGCGCACACCGATAAGGTGGGCCGCCTTTTGGCGGCATATGATGATATAGTTGTTGACGAGTTGCCGGACGAGTTGCCACCCAGCCGAGGGGTGGATCATGAGATTGAGACGGATGATTCGAAAAGGGCGCCCTTCCGGCGGCCCTACCGAATGACCCGGTACGAGTGGGCGGAGTTGGACAAACAGGTGAAGTCGCTCTTGGCCCGAGGTGTCATCAGGGAGTCAAAGTCACCTTTTGGAGCCCCGGTGCTGTTTATCAAGAAGAAGACTGGTGAGTTACGGATGTGCGTGGATTACAGGGCGTTGAACGACATGACGGTGAAGAACAGATACCCACTACCCCGAATCGATGATATCTTGGACACTCTGAACGGTGCAGTGGTCTTTTCTAAGCTGGATTTGCATTCAGGATATCATCAGGTGCGTATCAGAGAGGAGGACATTCACAAGACGGCTTTCACGACCAAGTCAGGTCACTATGAGTACATGGTGATGCCGTTCGGTCTGTGCAACGCACCCTCGACATTTCAGAAAATGATGAACGACACCCTGAAGCCGTTCCTGGACAAGACGGTCTGTGTCTATCTCGACGACATTATTATCTTCAGCAAGGACGTGGAGTCCCACCAGAAACACGTGGAGGAGGTGCTTGATCGACTGAGGGAGCAGAAGTTCTATGCCAAGAAGTCGAAATGCGAGTTCTTCAAGGATAGGATGGAGTTTTTGGGACATGTGGTCTCAGCAGAAGGCATTGAGGCCTGCCCAGAGAAAGTCAAGGCCGTGGAGGAATGGCCCCAACCCGAGTCTGGACTGAACTTGATGAGTTTCCTCGGGTTGGCCGGGTACTATCGACGGTTCATACCGAACTACTCACGCATCGCGAGCCCGTTGATAGAGTTGGCATCGGTGTCAACCAAAACTAAGAGGTCGGCCAAACCAGCGAGACCGTTCCTCTGGACACCGGAGTGCACGAAGGCCTTCCAGAAGGTCAAGGAGGAGTTGGTGTCAGGCAAAGTCATGATGATACCGACGATGGAGGACCCGTTCAAGGTGAGCACGGATGCTTGCGACGTTGCAGTAGGAGCGGTGCTGCAGCAGTGGAGTGTGAAGGATAAGGCGTGGCGACCGGTAGCGTACGAGTCTGCGAAGCTGACGGCCGCCCAGAAGAACTACTGCACGAGAGAGAAGGAGTTCTACGGGATTATCCATGCGTTGAAGAAGTGGAGACACTACCTGTTGGGACAGCCCTTCCGGATCGAGACGGACCACCAGTCACTGACGTACTTCTCGTCACAGACGGAGCCCCCGAGTGGAAGACTGAGTAGGTGGTTGGATTTTCTGGCAGACTATGACTTTGACATTAAGTACCTGCCAGGGGAGCAAAATGGAGCGGCGGACGCACTGTCGAGGATGACGGTGATGCCGGTGTGGTTTGCAGAGTCGGAAGAGGACGAGGGAGCATTGAAGGTGATGCCTGTGTGGGTAGAAGATGAGGAGATACGCGAGTTGGTGAAGAGGGGTTACAAGGACGACAAGGATTTTGGGGAGATTTGGGACATTTTGGTAAACGACAAGCCGGTACCGAAGACGATGGTGGAACACATTCGACATTTCAGCATCGACGAGAACAAGTTGCTGTATTTCGAGACGATTCCAGAAGGGGGAGAGGGAAAGCGAATGTGTATTCCAGCAGGCTTGCCGAGAGAGAGGATGAAATTGGAGGCACACGACACCCCGACAGCTGGCCACTTTGGCTACTACAAGACGTTCGACAGACTGTCCAGGAATTGTTACTGGCCCAGGATGATTAAGGAGATGCGTGAATACACGAAGTCGTGTGATGTCTGCCAGAGGACCAAGTCTCAGACAACGATGAAACAAGGCCTGCTGAAGCAGCTGCCAGTGCCGACGAGAAACTGGTCGGACATCTCAATGGATTTTGTGTCTAAGTCGGGTCTGACGCAGTCGCGTAACGGGTTCGACAACGTGTGGGTAGTTGTGGACCGCATGTCGAAGCAAGTGCATTTGATCCCGTGCCATATTGGAATTAATGCAGAAGAGACAGCAAATCTGTACCTGGACAGGATCTTCCGGTACCACGGAGTGCCTAAGACTATCGTGTCGGATCGGGATGTGAAGTTTGCCTCGAAGATGTGGCGTACGTTTCAGCACAGACTAGGAACGAAGCTGAAGTTCTCCACGGCAAACCACCCCGAGACCGATGGTCAGACGGAACGAGTGAACAGAGATGTGAGGCGGTTACTACGAACGTATGTGCAGACCAGACCTGACGAATGGGACGAATGGTTACCAGTGATGGAGTTCGCCATCAACTCGGCCGTGCACAGCACGACAGGGTACTCCCCGTTCGAGGTCAACTACGGGTTCGTGCCAGATGGACCGGCCTACGAATCGACCTTTATGATGGACCGGCCGCACCACCAGATGGACGAGTGGTTGGAGAAGATGAAGACGGTCCAAGGAGAAGTTAGGGACAGGATCGTCGAGATGCAAGGGGCGCAAGAGGCTCGAGTGAACCAGCACCGACGAGACGTGTCGGTCAAGGTGGGAGACATGGTGTTGGTTCATCGAAAGGCGTACTACAACAAGGGCGAGGACTCGAAGATGCATGATGTGTTCTTCGGGCCTTACCGGGCCCTGAAGCAGGTGTACGACAACGCATTCGAGGTGGCGTTGCCTCCCGAGTCCAAAAGACACCGGAACATCAACGTGCAGTTCTTAAAGAAGTATGAGGAGCGAGATGAGTACCTGTCCCAGCCACCGGTGCACGAGGAGCAACAGAGAGCCAACATCCACACTATCGTTCGGTTTGCCGGTATGGACCGAGAGAACGAGGAGGTGTTGTGCACGTGGGAAGGCTGCGACCCGCTGATCGCTACGCCAGTGCCGAGAGCACTGCTGGAAGAGTGCATGGATCCAGCGCGATTGGAGCAGCTGACAGAAGACTGGCTGCGTTGGGAGAAATTCCACGAGGAGGTCGAACGTGAGGAGCAAGCTGAGCTGCAGGAGGTTATGGTTGAACACCCTGGCAGCGTCGAGGAGGAGGACGACGAGGACCACGAGGACCACGTGACCGAGTCTTAAGGAGGGGAGAGTGTAAGGACCAAGTCGCTTACTAAGGGTTGGGACGGTGCCGGTCACCGTGCAAGGGTGACTGTTTTTCCCACCTGGGACGCACCCGGAGGATGGGAGTATAAAAAGGACATTTATGTATGATTAAGAAGACAAGTTATCACCAGCCCTGTGAACGTATTAACAGACAGCGAGCAACGGTTTAGATTAGACAGCGACAATCTACCGACTGAGAACTATTCCGCAGATAAGAAACGATCGGATCTTACAATTTTGCAACGGACTAAATCGACTCATAAGGTGATATAGGATTAGTCATGATCTGAGTTAAACTGCTGACTAAACAGCATCTGGGTTCTGACATGATTTGAAAGGTTTTGAAACTAAAAGTATGAAAAGAGGGATTATTTCGCAGATACGACGAAACTTGGACCATTATTGTATTATTTGGGGCTTTTAATTTCATATAAATCCTTCAAACCAACGATTTATGTGGATTTAGGGCACACCCGACCTTTTTGAAAAATCGTGACTTTTGCAACTTTTAATGACTTCTTGTGAGTCCGTCAAAGTCAAAAAAACTGTCAGAAACTGCGCCCAATCCATCTGTTTCGAAAAATTGACTCTACAACTACTCATATTTGCAGCTTGGTCTCATTTCTCTGGTTTGCCGCTCAAATCGGCTGTCGGAAGTTTCTGGGAACCTGCTGAGGCAGAAAACGTGTGAATTTGACGGGGAAAAGTTGAAAACTACCATTGTTGTGTTCCCTGGAGAGTTCTGGACAAGTCTTGGTTCAGGAATGTGGTCACCTTGTCACAATTATCTACAATACCAGTACTGTACAGTACATACTTGTATCCCCCCTCCATTTATCTCCTCCAACACTCTCAACAGACTTGTACAACTTGAAGTGATAATGTACTTGTTTTTGAACAATGAGACGTCCAGAGACCCGAAGAGACTATCAATTTACTCAAACCCACCACGTGACAGTTTTATCACCAGAAATCAACACCTTTTGCCAACACTCTGTATACGTCTTCTCAAAATAATCATACATATCTTAATTCACACCGACCAAAAAGTGCAGAAATACAAAGACCTATGAAACACGATAGAAATCGGATATATCGAAGAAGAAACAGCTTTATGCATGTCTAGATCATGCCACCATCCCA

Solo LTRs:

YALI0C: 1236840 – 1237083 (rev comp) no tRNA detected

CTGAAGTGCGCAATGACATTCGTTGTTCGCTTGTTCACGTAATTTTTTTTTTTTTAATTTTATTATTTTAATGAATGCTTTCTTGTCTTTTTTTAATATTATTATTATTCTAATGATTCCGCGCACATGACTTATTTGGCCCTTCAAAACGTCCTACGCTCTTTTCTTTCTCTGTAAGATCCGATCGCCTCTTATCTGCGGAATAGTTCTCAGTCGGTAGATTGTCGCTGTCTAATCTAACCGTTGCTCGCTGTCTGTAATACGTTCACAGGCTGGTGATAACTTGTCTTATTCATCATACATAAATGTCCTTCTTATACTACCATCCTCCGGGTGGGTCCCAGTTGGGAGCCACAGTCACACCTGCACGTGACCGGCACCATCCCAAGCCCTTAGTAAGCGACTTGGTCCTTACATTCTCAACGGTTGCGGC

YALI0A: 1751023 – 1751266

CAGAATCTGGAAAATCGCATGTAAGGACCAAGTCGCTTACTAAGGGCTTGGGACGGTGCCGGTCACGTGCAAGGGTGACTGTTGTTCCCACCTGGGACGCACCCGGAGGATGGGAGTATAAGAAGGACATTTATGTATGATTAAGAAGACAAGTTATCACCAGCCTGTGAACGTATTACAGACAGCGAGCAACGGTTAGATTAGACAGCGACAATCTACCGACTGAGAACTATTCCGCAGATAAGAGACGATCGGATCTTACATCGCATAAATCCGATATAGTGTAGGGGTTATCACTTCCGCCTTTCACGCGGAGAACCCGGGTTCGATTCCCGGTATCGGAGGTTCTTTTTTGGTCTCGAAAGGGACCGTGGCCGATTTTTTGGAATAATGAGGATACTGGAGTGGATATTTTGTGGTCGGGATTTTTACGAGTAAAAATACGGGAAATTTGGAGCGATTCCAGGCGGTTCGTTTGCTTTGTCGAGATATCTGCCATATGTCGAGTCT

Yali0E: 878636 – 878871 (rev comp) no tRNA detected

AAGGTTGAGATAACTGGGATTGATATGCAACCTTATCTACGATATAAAAAAATATGATCTGTGGTGACTTGCCAATATGAGGACTTGATACAGCCATGGGTAGGTAGAAAAAAAAATACCAGATCATTGTGATCACAAAAGGAGTGGACGACACCGGAATCGAACCGGGGACCTCGCGCCATGCTAAAGCGCATGTGATAACCAACTACACCAGATATAAGATTCCGATCGTCTCTTATCTGCGGAATATTCTCAGTCGGTAGATTGTCCGCTGTCTTAATCTAACCGTTGCTCGCTGTCTGTATACGTTCACAGGTGGTGATCACTTTTTTAATCATACATAAATGTCTTCTTATATACTCCCCCCCGGGTGCGTCCCAGGGGGGAAAACAGTCACCCTTGACGTGACCCGGCACCGTCCCCAAGCCCTTATAAGCGACTTTGGTCCTTACACCAGACGCCCAAGT

YALI0F: 1157244 – 1156975 (rev comp)

GACGCGATATGTCAACAGCCGAAATTTGACTTTTTTTATGTAAGATCCGATCGTCTCTTATCTGCGGAATAGTTCTCAGTCGGTAGATTGTCGCTGTCTAACCTAACCGTTGCTCGCTGTCTGTAATACGTTCACAGGCTGGTGATAACTTGTCTTATTAATCATACATAAATGTCCTTCTTATACTACCATCCTCCGGGTGGGTCCCAGTTGGGAGCCACAGTCACACATGCACGTTTTTTTTTTTTATTCTAGTGATTCGTGACCGGCACCATCCCAAGCCCTTAGTAAGCGACTTGGTCCTTACATTTTAGCTTTCCGATATAGTGTAGGGGTTATCACTTCCGCTTTTCACGCGGAGAACCCGGGTTCGATTCCCGGTATCGGAGATTCCTTTTTTTGATCGAGCAACGATTTATATCCTTCTTGCTCCAGCTATTTTTTTGCTTGGTTGCTCGTTTTCCATACTGCTGTTGCTTAATTTGAGGTTATCACTATACTACCTACAGTACAGACACACCTGCCTGTGTCTGAGGTGTACGCCCATTCTACGTAGGTACATACTGTACACGCGTGCAGTGTTATAATTCATTCAAATATCGTCGGGCTAGCCGCCAAT
